# Comparative Analysis of Chemical Distribution Models for Quantitative In Vitro to In Vivo Extrapolation

**DOI:** 10.3390/toxics13060439

**Published:** 2025-05-26

**Authors:** Hsing-Chieh Lin, Lucie C. Ford, Ivan Rusyn, Weihsueh A. Chiu

**Affiliations:** Department of Veterinary Physiology and Pharmacology, College of Veterinary Medicine and Biomedical Sciences, Texas A&M University, College Station, TX 77843, USA; hclin@tamu.edu (H.-C.L.); lucie.ford@tamu.edu (L.C.F.); irusyn@tamu.edu (I.R.)

**Keywords:** mass balance models, free concentration, in vitro assays, QIVIVE, bioavailable concentration

## Abstract

Quantitative in vitro to in vivo extrapolation (QIVIVE) utilizes in vitro data to predict in vivo toxicity. However, there may be differences between reported nominal concentrations and the biologically effective free concentrations in media or cells. This study evaluated the performance of four in vitro mass balance models for predicting free media or cellular concentrations. Comparing model predictions to experimentally measured values for a wide range of chemicals and test systems, we found that predictions of media concentrations were more accurate than those for cells, and that the Armitage model had slightly better performance overall. Through sensitivity analyses, we found that chemical property-related parameters were most influential for media predictions, while cell-related parameters were also important for cellular predictions. Assessing the impact of these models on QIVIVE accuracy for a small dataset of 15 chemicals with both in vitro and regulatory in vivo points-of-departure, we found that incorporating in vitro and in vivo bioavailability resulted in at best modest improvements to in vitro–in vivo concordance. Based on these results, we conclude that a reasonable first-line approach for incorporating in vitro bioavailability into QIVIVE would be to use the Armitage model to predict media concentrations, while prioritizing accurate chemical property data as input parameters.

## 1. Introduction

The Toxicity Testing in the 21st Century (Tox21) program, launched in 2007, addresses the growing need for risk assessment of thousands of chemicals [1]. Tox21 and related chemical testing initiatives aim to enhance the efficiency and accuracy of safety evaluations by leveraging advanced methodologies, including in vitro high-throughput screening (HTS) technologies and computational models [2,3]. In the past decade, the data availability from in vitro bioassays has increased dramatically [4,5]. As a result, there is a pressing need for methods that can extrapolate concentrations at molecular targets observed in in vitro bioassays, termed biologically effective doses (BEDs), to equivalent in vivo effective doses [6,7].

Quantitative in vitro to in vivo extrapolation (QIVIVE) has been proposed as a method to convert concentrations that produce adverse outcomes in vitro to corresponding in vivo doses using physiologically based kinetic (PBK) modeling-based reverse dosimetry [8,9,10]. Accurate linkage between in vivo and in vitro BED is crucial for improving QIVIVE. In vivo BED is typically represented by the concentration of free-dissolved chemicals in plasma or concentration of chemicals accumulated in tissue; concomitantly, the adverse effects are attributed to the amount of the chemical that can enter cells [11]. Similarly, in vitro BED is represented by the concentration of freely dissolved chemicals in media, the amount that is available for cellular uptake and effect [12]. However, a significant challenge in applying QIVIVE arises from the common use of “nominal” chemical concentrations that are reported for in vitro assays—these are not directly comparable to the “free” chemical concentrations in plasma observed in vivo [13]. Nominal concentration, defined as the total mass of a chemical added to a defined volume of the exposure medium, has traditionally been used to develop in vitro dose–response profiles; however, this approach has been criticized as not accurately reflecting in vivo BED due to differences in biokinetics. Consequently, it has been suggested that the freely dissolved concentration in media is a more appropriate metric for comparisons with the freely dissolved concentration in plasma from in vivo testing [14,15,16]. Although this notion has been proposed, only a few studies have considered chemical distribution (e.g., protein binding) in vitro and in vivo while conducting QIVIVE [17,18].

Measuring free concentrations in media typically requires additional chemical analyses, which, under the high-throughput demands of Tox21, can be both costly and technically challenging, particularly with small media volumes in HTS assays [19]. To address this limitation, mathematical models have been developed to characterize chemical distribution and predict cellular and free chemical concentrations. Proença et al. [13] provided a comprehensive review of various in silico models that describe chemical distribution in in vitro systems, detailing various aspects of each model, such as partitioning, as well as chemical- and in vitro test system-related domains of applicability. Specifically, in vitro distribution is governed by a chemical’s interaction with various compartments, including media constituents, the extracellular matrix, test system materials, intra-cellular accumulation, volatilization, and abiotic degradation. Regression-based approaches are often employed to estimate partition coefficients, such as the octanol–water partition coefficient (K_OW_), to derive equations for predicting chemical distribution [20,21].

The complexity and parameter requirements of existing in vitro mass balance models vary. For example, some models are designed for specific bioassays or chemical classes, such as the model developed by Gülden and Seibert [12], which is tailored for the experiments in glass Erlenmeyer flasks. This test system is not widely used anymore, especially for HTS assays. Conversely, other models, such as those by Fisher et al. [16], Armitage et al. [19], Fischer et al. [22], and Zaldivar-Comenges et al. [23], are applicable to various types of multi-well plates and a broad range of neutral and ionized organic chemicals. Inputs for these models require not only chemical property-related parameters to estimate partitioning coefficients, but also cell-, media-, and labware-related parameters. The availability of cell- and media-related parameters may be a limiting factor because many new cell types, such as induced pluripotent stem cells (iPSCs), may be used for HTS assays and their cell culture media composition may also be proprietary.

**Figure 1:**
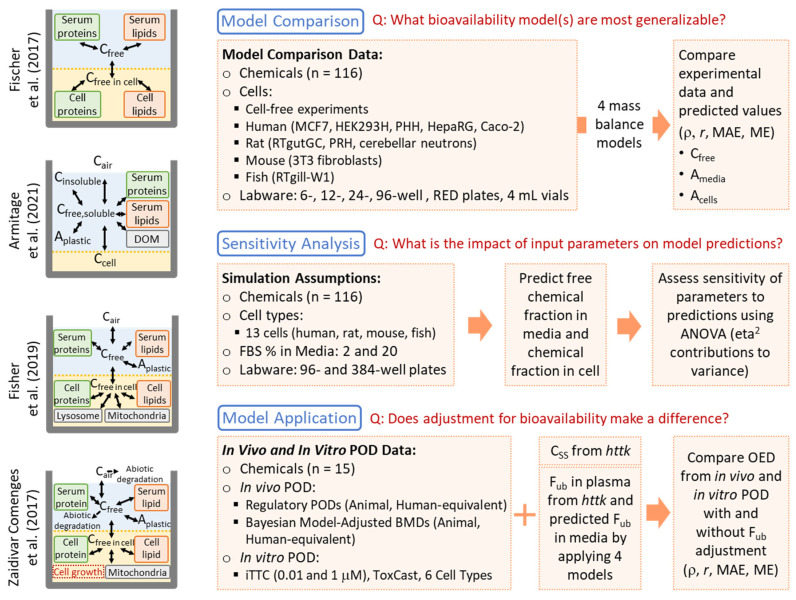
Schematic of study workflow and the structure of the four selected in vitro mass balance models [16,19,22,23].

A comparison of chemical distribution simulations and predicted cellular concentrations across five models for six chemicals, conducted by Proença et al. [13], revealed varying degrees of concordance between models. Understanding model performance in predicting cellular and free media concentrations is critical for QIVIVE; however, few mass balance models have been compared to experimental data. Among the four generic models listed above, only Armitage et al. [19] directly compared predictions with in vitro experimental data on chemical or cellular concentrations. Therefore, this study aimed to address three knowledge gaps in the field of in vitro mass balance models (Figure 1). First, we determined which models exhibit the best predictive performance by comparing their accuracy using a common experimental dataset that includes free fractions/amounts in media and cells. Second, we used sensitivity analysis to assess the impact of chemical-, cell-, media-, and labware-related parameters on model predictions, identifying the most important data to prioritize in terms of accuracy and quality. Third, we evaluated the extent to which including these in vitro and in vivo chemical distribution predictions in QIVIVE improves the in vitro–in vivo concordance of points of departure (PODs). Based on these results, we provide practical recommendations for the use of in vitro mass balance models and offer empirical evidence on how adjustments for in vitro and in vivo bioavailability may impact QIVIVE.

## 2. Materials and Methods

The overall workflow of this study is illustrated in Figure 1. It is divided into three main components: model comparison and performance evaluation, sensitivity analysis, and model application.

### 2.1. Model Comparison and Performance Evaluation

#### 2.1.1. Models Selected for Analysis

The recent comprehensive review by Proença et al. [13] compared the features and applicability domains of fourteen in vitro mass balance models. Some of these models are limited to specific chemical groups or cell types, while others have broader applicability across a wide range of chemicals and in vitro systems. To maximize the utility and generalizability of our analyses, we selected four existing chemical distribution models with broad applicability to chemical types and in vitro systems, as identified in the review: Fischer et al. [22], Armitage et al. [19], Fisher et al. [16], and Zaldivar-Comenges et al. [23]. Figure 1 includes a schematic of each model, and Table 1 provides a summary of their characteristics. All four models can be applied to a generic in vitro system. The first three models are suitable for systems exposed to both neutral and ionizable organic chemicals (IOCs), while the Zaldivar-Comenges et al. [23] model is only applicable to neutral chemicals. Except for the model by Fischer et al. [22], which only includes the media and cell compartments, the other three models were developed with consideration of the media/serum, cellular, labware, and headspace compartments. Individual models incorporate additional factors: Armitage et al. [19] include media solubility; Fisher et al. [16] account for cellular metabolism; and Zaldivar-Comenges et al. [23] incorporate abiotic degradation and cell number variation. Fischer et al. [22] and Armitage et al. [19] models are equilibrium partitioning-based models, while Fisher et al. (2019) [16] and Zaldivar-Comenges et al. [23] models simulate time-dependent concentrations. Detailed equations are provided in the respective publications.

For the Fischer et al. [22] model, the same authors published an updated model [21] incorporating distribution to plastic. However, this updated model focuses solely on the chemical distribution in media and plastic, without considering cells. As such, this model is limited to applications in cell-free assays and cannot be used to predict free concentrations in media that contain cells. Considering the model’s applicable domain, although an updated model [21] exists, we still used Fischer et al.’s [22] model.

Based on the chemicals applicability domain and considered compartments, each model requires different chemical parameters, listed in Table 2. Common parameters include molecular weight (MW), melting point (MP), and octanol–water partition coefficient (K_OW_). The models by Fischer et al. [22], Armitage et al. [19], and Fisher et al. [16] also include pKa and IOC types as key input parameters. For models that consider the headspace compartments, such as those by Armitage et al. [19], Fisher et al. [16], and Zaldivar-Comenges et al. [23], additional parameters such as the air–water partition coefficient (K_AW_), molecular volume (Vb), and Henry’s constant at 37 °C (H37) are needed. Also, Armitage et al. [19] incorporate solubility into the model, meaning solubility and the salting-out constant (K_salt_) are needed. It is noted that the model by Fischer et al. [22] has a different parameterization, requiring the distribution ratios at pH 7.4 between bovine serum albumin (BSA)/phospholipid liposomes (lip) and water (D_BSA/w_ and D_lip/w_).

#### 2.1.2. Data Used for Comparisons

The datasets were selected for model comparisons because they provide experimental measurements for at least one partitioning compartment relevant to the model structure, along with sufficiently detailed information on the experimental protocols to allow for an accurate implementation of model simulations. Each dataset is briefly described below in terms of the measurement type, chemicals tested, cell types, and other experimental details (summarized in Table 3). Additional details are available in Table S1.

(1) Ratio of free to nominal concentration

Three of the datasets that reported free fraction in media were sourced from Armitage et al. [19], who collected data from three previous publications [24,25,26]. Data from Huchthausen et al. [24] included 12 chemicals and were calculated by dividing the measured freely dissolved IC10 by the nominal concentration. Tanneberger et al. [25] and Schug et al. [26] reported ratios of free concentration in media after 24 h to the initial nominal concentration (C24/C0) for 27 and 9 chemicals, respectively. Data from these studies were obtained directly from Armitage and colleagues.

Additional data from Nicol et al. [27] was also included, where the bound fractions were calculated from the measured concentrations in buffer and total tested media from three different cell types (HepG2, HepRG, and MCF7). For each media type, 30 chemicals were tested at nominal concentrations of 10 µM using Rapid Equilibrium Dialysis (RED). The reported results ranged from 10% to 99% bound, with values outside this range reported as <10% or >99% bound. The unbound fraction was calculated as 100% minus %bound, with the censored %bound values of <10% and >99% replaced by 5% (the midpoint between 0% and 10%) and 99.5% (the midpoint between 99% and 100%), respectively.

From our laboratory, data on media-to-nominal concentration ratios for 10 drugs [29], 20 pesticides [28], and 14 PFAS (see Analytical Method description in Supplementary Materials) were included. The media used for these protein-binding assays consisted of iCell Cardiomyocytes maintenance medium (FujiFilm Cellular Dynamics, Madison, WI, USA), which contains 5% or 10% fetal bovine serum (FBS) depending on the study. The fraction unbound was calculated from concentration data (free and stock). The details of chemical exposure and process of LC-MS/MS analysis are detailed in the work of Blanchette et al. [29] for drugs, Valdiviezo et al. [28] for pesticides, and Supplementary Materials for PFAS. Blanchette et al. [29] include data for only one exposure concentration; meanwhile, the other two studies provide data at two exposure concentrations, so for these studies each chemical has two datapoints.

Overall, this dataset includes 269 observations of 116 unique chemicals, with several chemicals, including Caffeine, Lamotrigine, Ibuprofen, 2,4-Dinitrophenol, Chlorpyrifos, Disulfoton, Lindane, Parathion, and Chlorpromazine, tested in multiple studies. All observations are listed in Table S4.

(2) Chemical amount in media and in cells

Data on the amount of chemicals in cells and media comprise data from studies that included chemical concentrations in cell lysates and/or media in each experimental well. Some studies [30,32,34,35] added fresh chemicals to cell cultures daily for 14 days, and data were reported for Day 0 and Day 13, with multiple timepoints. For consistency, we only used data collected from within the first day (≤24 h). Notably, Kodavanti et al. [33] exposed cells for 60 min with sampling at 15, 30, and 60 min, and Broeders et al. [31] sampled at various timepoints (48 h for Balb/c 3T3 and Caco-2 cells, or 72 h for HepaRG cells). Where tabulated data were not available, values were extracted from figures using WebPlotDigitizer version 4 (https://apps.automeris.io/wpd4/) (accessed on 2 April 2024). Overall, there are 27 and 43 datapoints for chemical amounts in media and cells, respectively, for 7 unique chemicals, with some studies using multiple timepoints or experimental replicates (Table S4).

#### 2.1.3. Model Parametrization

Parameters required for the in vitro mass balance models were categorized into the following types: chemical, cell, media, and labware. Details for each category are described below.

(1) Chemical-related parameters

**Figure 2:**
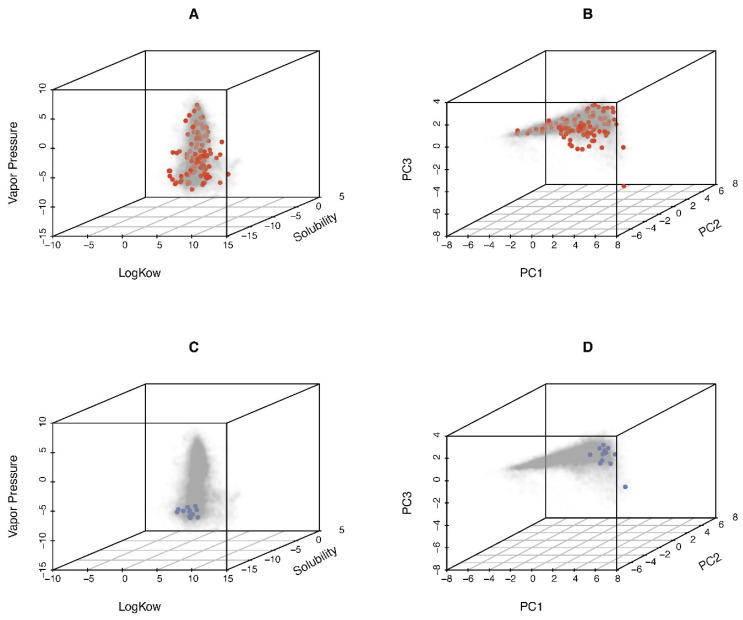
Characteristics of unique 116 chemicals used to model performance comparison (red points, (**A**,**B**)) and 15 chemicals used to model application (blue points (**C**,**D**)), compared to the chemical space included in the Collaborative Estrogen Receptor Activity Prediction Project (CERAPP; gray points; *n* = 24,955). (**A**,**C**) Distributions of log K_OW_, solubility, and vaper pressure. (**B**,**D**) Principal component analysis (PCA) plot demonstrating the diversity of study chemicals.

Based on the above-mentioned details of the dataset, our whole dataset included 116 unique chemicals. To illustrate the diversity of the studied chemicals, we compared our 116 chemicals to those in the Collaborative Estrogen Receptor Activity Prediction Project [36] (CERAPP; *n* = 24,955). Nine chemical descriptors, including molecular weight, boiling point, Henry’s law constant, melting point, octanol–air partition coefficient, octanol–water distribution coefficient, octanol–water partition coefficient, vapor pressure, and water solubility, were sourced from the Integrated Chemicals Environment (https://ice.ntp.niehs.nih.gov/) (accessed on 21 January 2025) (Table S5) and analyzed using principal component analysis (PCA). Plots showing chemical space coverage for octanol–air partition coefficient, vapor pressure, and water solubility, as well as the first three PCs, are presented in Figure 2 (corresponding two-paired scatter plots in Figure S1). The results demonstrate that our 116 chemicals are largely representative of the structural diversity of the CERAPP chemicals.

For consistency of parameterization, most of parameters, i.e., MW, MP, K_OW_, K_AW_, solubility, and K_salt_ were sourced from the Exposure and Safety Estimation (EAS-E) Suite platform (ARC Arnot Research and Consulting, https://arnotresearch.com/eas-e-suite/) (accessed on 24 April 2025). These parameter values are compiled in Table S2. For pKa and IOC identification, we used the website (https://xundrug.cn/molgpka) (accessed on 20 January 2025) developed by Pan et al. [37] to predict the pKa and determine the IOC type (Table S6). Some chemicals may have multiple pKa values, each corresponding to different acid or base types, and the process for identifying which pKa and IOC to use involves first determining the strongest acidic or basic pKa (if applicable). Then, the pKa values are compared to the pH value of the in vitro system (7.4). If an acidic pKa is greater than the system pH by 2 or more log units, it is considered predominantly neutral and can be ignored. The same principle applies for basic pKa values: if the pKa is more than 2 log units lower than the system pH, the compound is considered neutral. The final determined pKa values and IOC types based on this procedure are in Table S2. Additional parameters for Zaldivar-Comenges et al. [23] include molecular volume (Vb) and Henry’s constant at 37 °C (H37), and these values were obtained from the US EPA CompTox Chemicals Dashboard (https://comptox.epa.gov/dashboard/) (accessed on 24 April 2025). Fischer et al. [22] required D_BSA/w_ and D_lip/w_: preferred data were experimental values, with predicted values used when experimental data were unavailable. For neutral chemicals, these values were primarily predicted using polyparameter linear free energy relationships based on chemical descriptors from the UFZ-LSER database (http://www.ufz.de/lserd) (accessed on 24 April 2025), following the equations presented in Fischer et al. [22] However, if the descriptors from the UFZ-LSER database were unavailable, Fischer provided via personal communication valued based on the relationships between log K_OW_ and D_BSA/w_/D_lip/w_ developed by Endo and Goss [38] and Endo et al. [39], respectively, were used to predict distribution ratios. For acidic and basic chemicals, the pKa was used to correct the log K_OW_, which was then used as input to predict the D_BSA/w_ and D_lip/w_. The detailed calculations and parameter sources were compiled in Table S7.

(2) Cell-, media-, and labware-related parameters

Information on these parameters was obtained, where available, from original references listed in Table S1, with specific values including cell types, cell seeding numbers, exposure concentrations, media volume, serum percentage, and labware types. Table S3 compiles the cell-related information, including size, mass, percentage of protein, storage, and membrane lipid and water content. Table S8 contains the labware dimensions. For parameters that were not reported, default values for each model were applied.

#### 2.1.4. Model Assumptions, Execution, and Evaluation

Based on the model applicability domain, models by Fischer et al. [22], Armitage et al. [19], and Fisher et al. [16] can be applied for predicting the in vitro distributions of all chemicals in our dataset, while the model by Zaldivar-Comenges et al. [23] can only be used for neutral chemicals. For Fisher et al. [16], we assumed no cellular metabolism. For Zaldivar-Comenges et al. [23], we assumed no abiotic degradation and no significant change in cell numbers. Model simulations for Fischer et al. [22] and Armitage et al. [19] were executed using Excel tools as published. The Excel tool for Fischer et al.’s [22] model was obtained from the Supplementary Materials of the original reference, while the Excel simulation tool of Armitage et al. [19] model was directly provided by Armitage and coworkers. Fisher et al. [16] model equations were coded in R language with GNU MCSim [40], and Zaldivar-Comenges et al. [23] equations were also implemented in R software, as sourced from Proença et al. [13]. Copies of all Excel sheets and R scripts are provided in https://github.com/hsingchiehlin/Invitro_mass_balance_models.

Model performance was evaluated using Pearson’s (*r*) and Spearman’s (*ρ*) correlation coefficients, mean absolute error (MAE), and mean error (ME). MAE was calculated to assess overall accuracy:

$$MAE=\frac{\sum \left|log10(Prediction)-log10(observation)\right|}{n},$$

while ME (calculated as the mean difference between log10-transformed prediction and observation) was used to assess the degree of bias. Linear regression analyses were employed to evaluate the impact of chemical properties (nominal concentrations, Henry’s constant [H37], molecular weight [MW], melting point [MP], solubility, octanol–water partition coefficient [K_OW_], air–water partition coefficient [K_AW_], and pKa) on prediction residuals, thus assessing whether there are limitations in the applicability domain of each mass balance model based on these chemical properties. Cyclosporine A was excluded from the regression analysis due to it having a much larger MW and smaller K_AW_ compared to the other chemicals.

### 2.2. Sensitivity Analysis

Sensitivity analysis was performed to examine the impact of model type and input parameters (chemical, cell, labware, and media) on predictions across different mass balance models. Although each category of inputs possesses several detailed parameters, to simplify the analysis, we combined some of the parameters based on the category. The analysis involved using the four selected in vitro mass balance models to predict the fractions of free chemicals in media and chemicals in cells, based on 1 μM exposure of 116 chemicals to 13 cell types in 96- or 384-well plates with 2% or 20% FBS in the media (Figure 1). The list of 13 cell types included those used for model comparison and additional cell types from the EAS-E Suite platform. However, we also exclude the cell types if their cell parameters, such as the lipid and protein contents, were set to the same of values as default or similar cell types, resulting in a final list of 13 cell types including human, rat, mouse, and fish cells (Table S3). Cell numbers and media volumes followed the default settings from the EAS-E Suite platform: 20,000 cells and 150 μL media for 96-well plates, and 5600 cells and 40 μL media for 384-well plates. Given the applicability domain of the mass balance models, the Zaldivar-Comenges et al. [23] model is not suitable for the IOCs. Therefore, we conducted two sensitivity analyses: the first considered all chemicals, evaluating only the models by Fischer et al. [22], Armitage et al. [19], and Fisher et al. [16]; the second focused exclusively on neutral chemicals, allowing us to incorporate all four models in the sensitivity analysis. For statistical performance, eta squared (*η*^2^) was calculated based on the context of an analysis of variance (ANOVA) to assess the contributions to variance of each input variable. The equation for *η*^2^ is

$$\eta^{2}=\frac{{SS}_{Variable}}{{SS}_{Total}}$$

where *SS_Variable_* is the sums of squares for the given input variable, and *SS_Total_* is the total sums of squares for all variables. The *lsr* R package (ver. 0.5.2) was used to calculate *η*^2^.

### 2.3. Chemical Distribution Model Application: Assessing In Vitro–In Vivo Concordance of PODs

Adjusting for in vitro bioavailability has been hypothesized to improve the in vitro–in vivo concordance of oral equivalent dose (OED) estimation by using QIVIVE. To test this hypothesis, we extend the work of Lu et al. [41], who used a dataset of 15 chemicals to compare regulatory in vivo PODs with multiple alternative in vitro PODs. The unit of the in vivo POD reported in Lu et al. [41] is mg/kg/day, which represents an external exposure dose. Therefore, it can also be expressed as OED to allow for direct comparison with OEDs converted from in vitro PODs (µM). Specifically, in vivo PODs in the work by Lu et al. [41] included four types: regulatory in vivo PODs in terms of both animal dose and human-equivalent dose (HED), as well as two Bayesian model-averaged benchmark doses (BMA BMD) in animal dose and HEDs. The BMA BMDs were derived using a web-based Bayesian BMD (BBMD) system (https://benchmarkdose.org) from the in vivo original study data used for regulatory assessments. The HEDs were extrapolated from the animal dose PODs using allometrically scaling and exposure duration uncertainty factors, where applicable. Detailed values, extrapolation methods, and relevant study information are compiled in Table S9. For more information, please refer to Lu et al. [41].

In vitro datasets included the fifth percentile AC_50_ from ToxCast and the most sensitive POD from a 6-cell type battery of in vitro testing from Chen et al. [42] and Ford et al. [43]. The latter dataset included multiple cell types, i.e., human-induced pluripotent stem cell (iPSC)-derived cells (hepatocytes, neurons, cardiomyocytes, and endothelial cells), human umbilical vein endothelial cells, and lymphoblast cells. For these 15 chemicals (listed in Table S9), we evaluated their representativeness of chemical space relative to CERAPP using the same approach as for the 116 chemicals used in model performance evaluation and sensitivity analyses. As shown in Figure 2 and Figure S1, the coverage of chemical properties is less complete for these 15 chemicals.

Lu et al. [41] previously noted that the best concordance was between the Bayesian model-averaged benchmark dose adjusted to HED (BMA BMD_H_) for in vivo PODs and the six-cell type battery for in vitro PODs. Here, we extended the work of Lu et al. [41] by applying different mass balance models to see whether they improved in concordance between in vivo and in vitro PODs. In addition to the two sets of in vitro PODs evaluated previously (ToxCast and the six-cell type battery), we also evaluated concordance using fixed constant in vitro PODs corresponding to equal toxicodynamic bioactivity. In this way, we could isolate the effects of toxicokinetics (TK) on IVIVE predictions, and to compare the degree of in vitro–in vivo concordance from TK alone with and without accounting for bioavailability. We selected constant in vitro PODs of 0.01 μM or 1 μM based on the concept of internal Threshold of Toxicological Concern (iTTC), where the value of 0.01 μM is the internal NOAEL (i.e., iTTC prior to application of a 100-fold safety factor) as proposed by Arnot et al. [44], and the value of 1 μM was the iTTC itself (which had no safety factors) as proposed by Blackburn et al. [45]

To examine the concordance of OED estimation from in vivo and in vitro studies, in vitro PODs were first converted using the standard QIVIVE approach:

$$OED\left(\frac{\frac{mg}{kg}}{day}\right)={POD}_{in\;vitro}\left(\mu M\right)\;\times \frac{1\;mg/kg/day\;}{C_{ss,\;1\;mg/kg/day}(\mu M)}$$

where $C_{ss,\;1\;mg/kg/day}$ is the steady-state concentration in plasma under daily oral dosing of 1 mg/kg/day, calculated using the calc_analytic_css function with the “3compartmentss” model and the settings of “adjusted. Funbound.plasma = FALSE” and “adjusted. Clint = FALSE” in the httk R package (version 2.3.0). We note that the steady-state concentration (rather than C_max_) is most appropriate for extrapolating to OED in this case because the mass balance models are 24 h or equilibrium in vitro concentrations.

To evaluate the impact of mass balance models, the OED calculation was adjusted to incorporate the fraction unbound in plasma (*f_ub, plasma_*) and in media (*f_ub, media_*):

$${OED}_{adjusted}\left(\frac{\frac{mg}{kg}}{day}\right)=\left({POD}_{in\;vitro}\times f_{ub,\;media}\right)\;\times \frac{1\;mg/kg/day\;}{\left(C_{ss,\;1\;mg/kg/day}\times f_{ub,\;plasma}\right)}.$$

Here, *f_ub, plasma_* was sourced from the httk database and *f_ub, media_* was calculated using the four in vitro mass balance models described above. Due to the multitude of unknown cell-based parameters for ToxCast data, *f_ub, media_* predictions were based on the following default assumptions for cell, media, and labware parameters: the default cell type in the Armitage et al. [19] model, using 384-well plates with the hiPSC-CM maintaining media used in Chen et al. [42] (measured lipid content: 0.22 mg/mL; measured protein content: 4.19 mg/mL).

Chemical-related parameters are summarized in Table S2. Concordance was evaluated using *r*, ρ, MAE, and ME, as previously described. Because the BMA BMD_H_ in vivo PODs included confidence interval estimates, *r* and ρ were calculated weighted by the inverse variance, as implemented using the wCorr R package (version 1.9.8). The impact of mass balance models on concordance with respect to alternative in vivo POD types considered by Lu et al. [41] was evaluated in a sensitivity analysis.

## 3. Results

### 3.1. Comparing Performance of Four In Vitro Mass Balance Models

**Figure 3:**
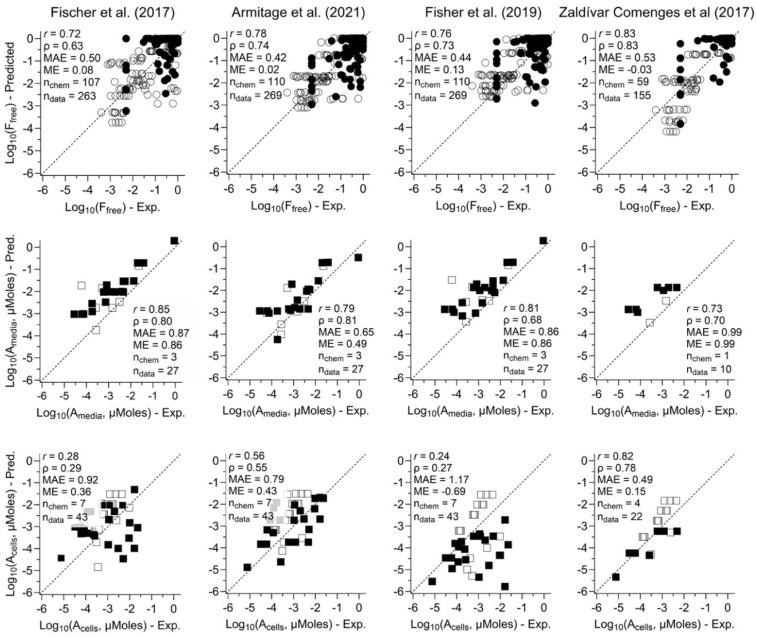
Scatter plots of comparing observed free fraction in media (ratios of free to nominal concentration) (**top row**), amounts of chemicals in media (**middle row**), or cells (**bottom row**) and their corresponding predictions by using four in vitro mass balance models (see column labels). For free fraction (**top row**), the filled points represent the data sourced from the literature, the open points represent experimental data from our previous publications. For amounts in media (**middle row**) or cells (**bottom row**), the filled points represent the experimental data tested using human cells, the open points represent experimental data tested using non-human cells. Each graph shows correlation values and prediction errors, as well as the number of chemicals (n_chem_) and data points (n_data_) included in each scatter plot. See Table S4 for the values plotted in these graphs [16,19,22,23].

The comparative performance of the four selected in vitro mass balance models in predicting the ratios of free to nominal concentration (F_free,media_), chemical amount in media (A_media_), and in cells (A_cell_) is illustrated in Figure 3 (residuals shown in Figure S2, separated by neutral and ionized). Additionally, because the Zaldivar-Comenges et al. [23] model only is applicable to neutral chemicals, we separated performance summaries for neutral and ionized chemicals in Figure S3. Nicol et al. [27] reported analytical issues for some measurements, so we also evaluated model performance excluding those data and found that it showed little differences in *r*, *ρ*, MAE, and ME (Figure S4).

For model comparison, we focus on MAE as the measure of prediction accuracy and ME as the measure of bias. Across the different types of measurements, the most accurate (lowest MAE) and least biased (ME closest to 0) predictions were for F_free,media_, with predictions for A_media_ and A_cell_ performing worse, with the highest MAE observed for predicting A_cell_ using Fisher et al.’s [16] model. In most cases, predicted F_free,media_ was nearly unbiased, with a high peak in residuals near 0 deviation (Figure S2). In contrast, most predicted A_media_ and A_cell_ were biased high (ME ranged from 0.15 to 0.99), which the exception of Fisher et al. [16], which had high underprediction bias for A_cell_ (ME = −0.69).

Across models, the Armitage et al. [19] model had the best performance for F_free,media_ and A_media_ and Zaldivar-Comenges et al. [23] the best for A_cell_, though the differences across models for F_free,media_ were small. These conclusions apply for all chemicals combined and for neutrals only; however, Zaldivar-Comenges et al. [23] is only applicable to neutral chemicals. For ionized chemicals, the remaining three models perform very similarly for F_free,media_ while Armitage et al. [19] performs best for A_media_ and A_cell_ (albeit data only available for two and three chemicals, respectively).

With respect to residual trends, for predictions in media, most models and parameters had no significant trends and/or coefficient of determination (R^2^) < 0.10 (10%), indicating that errors in most cases did not systematically shift with chemical properties. Exceptions were a significant positive trend with pKa in the Fischer et al. [22] model (R^2^ = 0.13), and the significant positive trend for log10_Solubility (R^2^ = 0.12) and negative trend for log K_OW_ (R^2^ = 0.19) in the Zaldivar-Comenges et al. [23] model (Table S10 and Figure S5). Thus, Fischer et al. [22] under/over-predict with low/high pKa, and Zaldivar-Comenges et al. [23] under/over-predict at low/high solubility and high/low-log K_OW_.

For predictions in cells, all four models had negative trends with log10_Solubility, but only for the Fischer et al. [22] model was the trend significant with a material R^2^ = 0.40 (Table S10 and Figure S6). Both Fischer et al. [22] and Fisher et al. [16] had significant positive trends with log10_H37, with relatively high R^2^ values of 0.29 and 0.72, respectively. The Zaldivar-Comenges et al. [23] model also had a positive trend with tested concentration, with high R^2^ = 0.58. The Fisher et al. [16] model also had positive trends with log K_AW_, log K_OW_, and MW, with R^2^ between 0.26 and 0.32. For the IOCs, our results found that three models which can be applied to IOC have negative trends for pKa with R^2^ values ranging from 0.12 to 0.26, meaning that these models tend to underpredict chemical distributions in cell at high pKa.

Overall, predictions in media have more consistent performance across different chemical properties than predictions in cells. Among models, for predictions in media, the Armitage et al. [19] and Fisher et al. [16] models had the fewest residual trends, with no chemical parameters having statistically significant trends and R^2^ > 0.1. For predictions in cells, only for the Armitage et al. [19] model were there no statistically significant trends and no R^2^ > 0.1

### 3.2. Determining the Impact of Input Parameters on In Vitro Bioavailability Predictions

**Figure 4:**
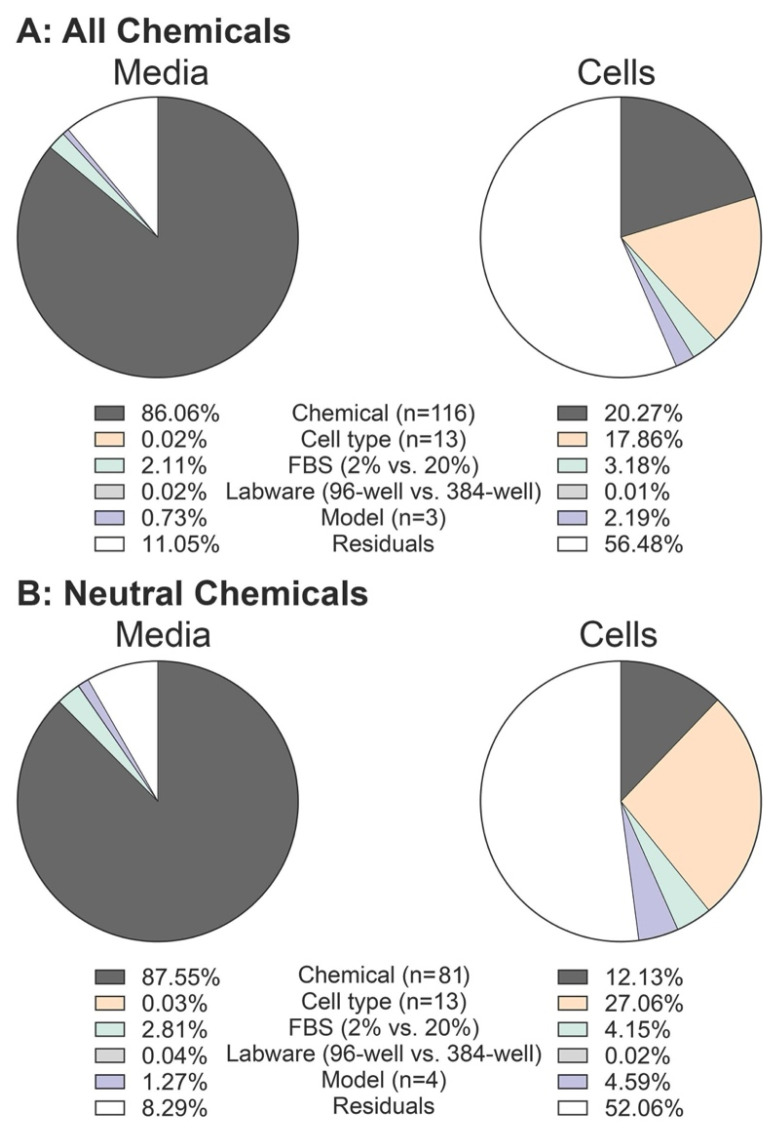
Pie charts showing the effect size (*η*^2^ values) for chemical, cell type, % of FBS, labware, and models on predicted chemical’s fraction in media (**left**) and cells (**right**). Calculations are shown for (**A**) all chemicals (*n* = 116) and (**B**) neutral chemicals (*n* = 81).

To evaluate the influence of different models and input parameters on chemical distribution predictions in in vitro assays, we conducted sensitivity analyses on F_free,media_, A_media_, and A_cell_ using the four mass balance models. The simulations involved 116 chemicals across 13 cell types in two types of labware with two serum concentrations. The results for ratios of free concentrations in media and chemical fractions in cells are shown in Figures S7 and S8, respectively. Eta squared (*η*^2^) values, derived from ANOVA analysis, illustrate the contribution of each variable to the variance, as presented in Figure 4, separately for all chemicals and neutrals only. For predicting F_free,media_, chemical-related parameters showed the highest sensitivity (*η*^2^ = 86.1% and 87.6%, all and neutral chemicals, respectively), followed by the percentage of FBS (2.1% and 2.8%, respectively). In contrast, for predicting A_cell_, cell-related (17.9% and 27.1%, respectively for all and neutral chemicals) and chemical parameters (20.3% and 12.1%, respectively) had the greatest influence. Different model types and labware showed very small sensitivity for either media or cells.

Additionally, to assess how concordant different models were with each other, we compared the predictions for each chemical and model from the sensitivity analysis to a “baseline” prediction from the Armitage et al. [19] model using the “default cell” settings (Figures S9 and S10). The results indicate that the Armitage et al. [19] model’s predictions for F_free,media_ are very similar to those of the Fisher et al. [16] model, whereas the Zaldivar-Comenges et al. [23] model generally predicts lower values. Predictions from the Fischer et al. [22] model exhibited the most notable differences, with some values (e.g., Diclofenac and Perfluorohexanesulfonic acid) being more than 10^2^-fold smaller, and others (e.g., 2,3-dimethyl-1,3-butadiene) being over 10^3^-fold larger than baseline values. For A_cell_, predictions from the Fisher et al. [16] and Zaldivar-Comenges et al. [23] models were generally lower compared to the Armitage et al. [19] model. Based on the box-and-whisker plot in Figure S10, many of the outliers with large discrepancies of A_cell_ between the Armitage et al. [19] and Fisher et al. [16] models were associated with two cell types: FishRtgill and rat cerebellar granule cells. These discrepancies may be attributed to cellular characteristics. The rat cerebellar granule cells, for instance, have an extremely high lipid content (95.3%), well above the range observed in other cell types (0.46–14.2%) (Table S3). Additionally, the FishRtgill cells have the lowest mass among the 13 cell types evaluated in this study. These differences likely influenced the variability in predicted chemical concentrations between the models.

### 3.3. Model Application: Effects of In Vitro Bioavailability Adjustment on In Vitro–In Vivo Concordance of POD Estimations

**Figure 5:**
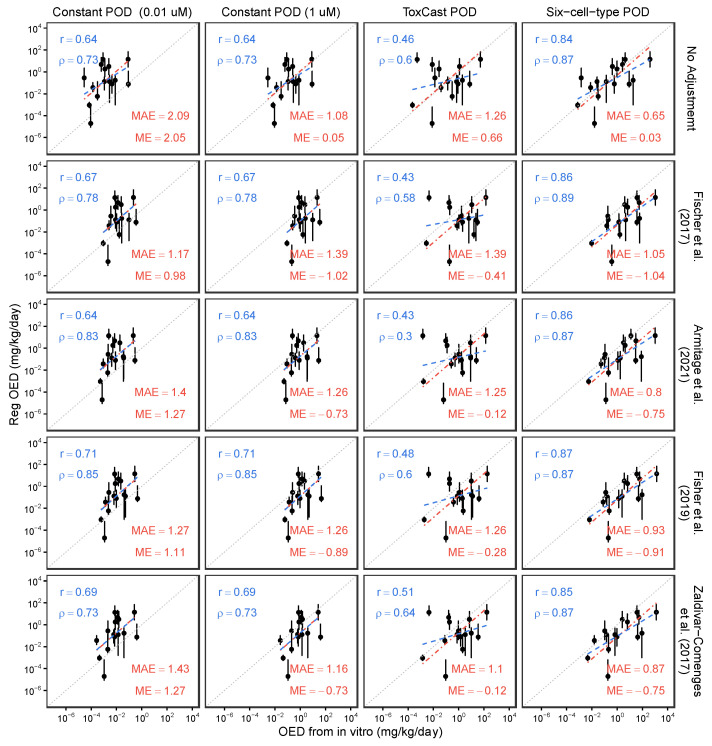
Scatter plots for comparing oral equivalent dose (OED) from in vivo and in vitro POD—(i) assumed to be constant (0.01 and 1 µM), sourced from (ii) ToxCast, and (iii) six-cell-type databases—with/without adjustment by using fraction unbound in plasma from httk database and fraction unbound in media predicted by four mass balance models. Blue dashed lines are best fit linear correlation (basis for Pearson *r* and Spearman *ρ* values), and red dash-dot lines are best fit line with fixed slope = 1 (basis for mean absolute error [MAE], and mean error [ME] values) [16,19,22,23].

Figure 5 illustrates the results of evaluating in vitro–in vivo concordance, with and without in vitro bioavailability adjustments, between the in vivo BMA BMD_H_ and the in vitro iTTC of 0.01 and 1 µM, the in vitro PODs from ToxCast, or the in vitro PODs from the six-cell type battery. In general, in vitro PODs from ToxCast generally exhibited lower concordance than those derived from either constant concentrations or the six-cell type dataset. Compared to using nominal concentrations alone, mass balance model-based adjustments for *f*_ub_ in plasma and media enhance in vitro–in vivo concordance for the constant iTTC of 0.01 µM, as evidenced by increased *r* and/or ρ values, decreased MAE, and ME values closer to zero. For the constant iTTC of 1 µM, there were also increases in *r* and/or ρ values, but MAE and ME did not improve. Improvements were less consistently observed with in vitro PODs from ToxCast. For the six-cell type battery, unadjusted concordance was already quite high, and although *r* and/or ρ values improved slightly with mass balance adjustments, MAE and ME tended to worsen. It is also noteworthy that the negative MEs mean that considering in vivo and in vivo bioavailability adjustments may make the OED derived from in vitro POD less conservative. Among the in vitro mass balance models, only small differences were noted, with no one model consistently leading to greater improvement in in vitro to in vivo concordance.

As a sensitivity analysis, similar analyses were performed with alternative in vivo PODs (Figure S11 and Table S11). Notably, in vitro–in vivo concordance with these alternative in vivo PODs from the ToxCast dataset remained lower compared to those derived from iTTC or the six-cell type battery, consistent with the findings from in vivo BMA BMD_H_. Although *f*_ub_ adjustments did not substantially enhance the in vitro–in vivo concordance of human-equivalent regulatory PODs (not using BMA) and the in vitro six-cell type battery PODs, in vitro–in vivo concordance using experimental animal PODs and constant and six-cell type battery in vitro PODs did improve with *f*_ub_ adjustments, with lower MAEs and ME values closer to zero.

To critically evaluate the quantitative impact of considering in vivo and in vitro bioavailability adjustments on in vitro–in vivo concordance, we calculated the “correction factors” that would be applied to nominal-concentration-based IVIVE, i.e., the ratio of fraction unbound in media to fraction unbound in plasma (Figure S12). In addition to the 15 chemicals evaluated for in vitro–in vivo concordance, we expanded the analysis to include a broader range of chemicals by using predicted fractions unbound in media from a sensitivity analysis and fraction unbound in plasma data sourced from the R package httk (version 2.3.0) dataset. The httk dataset contains fraction unbound values for 68 chemicals, and the predicted fractions unbound in media from human cell lines, including MCF7, HCT116, HEK293T, HEK293H, HepG2, Me 180, and SH-SY5Y, as well as the default cell, were used. Figure S12 demonstrates that most of these “correction factors” for in vivo and in vitro fraction unbound were almost all greater than 1, regardless of whether the 15 chemicals used in the model application or the 68 chemicals from the sensitivity analysis were considered. This finding suggests that, for most chemicals, incorporating both in vivo and in vitro bioavailability adjustments when performing QIVIVE leads to a higher, and thus less conservative, OED. Mechanistically, this is consistent with in vitro free fractions typically being higher than in vivo free fractions due to the higher protein content of plasma versus media.

## 4. Discussion

Proposals to use in vitro assays as a basis for human health assessments have resulted in the necessity for IVIVE approaches to convert in vitro bioactive concentrations to equivalent in vivo exposures. This conversion is crucial for accurately characterizing human health risks. Existing IVIVE methods often rely on nominal concentrations to derive an in vitro POD, which is then extrapolated with reverse toxicokinetics to an in vivo POD based on the total chemical concentrations in plasma. However, without incorporating biokinetics into in vitro systems, these approaches may introduce biases in assessing in vitro–in vivo concordance of effective concentrations causing any biological response. To address this issue, using free chemical concentrations in media or cellular environments has been proposed as a more appropriate approach for IVIVE. Nonetheless, measuring free concentrations, particularly in high-throughput screening assays, poses analytical challenges due to limited sample volumes and high numbers of samples. As a feasible alternative, in vitro mass balance modeling has been proposed.

Here, we evaluated the performance of four such in vitro mass balance models finding that predictions of media concentrations were more accurate than those for cells, and that the Armitage model had slightly better performance overall. We also found chemical property-related parameters to be most important for media predictions, while cell-related parameters were also important for cellular predictions. These findings aligns with previous studies that also found that predicted cell concentrations were less accurate than media concentrations [46]. It has been suggested that sample preparation techniques—such as the use of detergents, freezing and thawing cycles, ultrasonication, liquid homogenization, and PBS wash—can impact the “true” cellular concentration measurements, potentially increasing measurement variability and reducing robustness. Similarly, Bloch et al. [47] highlighted that the accuracy of partitioning data, along with cell-related parameters (e.g., cell mass, seeding density, and cell composition), are key factors influencing predictions of chemical distribution within cells. These factors could contribute to the observed biases in our predictions and offer potential methods for improving the accuracy of cellular predictions.

This analysis does have a number of limitations. The dataset for cellular chemical amounts was much smaller compared to that for media measurements. Additionally, parameterization across the four in vitro mass balance models varied and certain cell-related parameters for specific cell types, such as human and rat primary hepatocytes, were often unavailable. Consequently, default parameters were used, or parameters for other cell types were used as a surrogate. Furthermore, we could not test the performance of certain model components, such as cell growth and abiotic degradation in the Zaldivar-Comenges et al. [23] model and metabolism in the Fisher et al. [16] model, due to lack of appropriate validation data. For the sensitivity analysis, limitations were also present. Interaction effects within groups of cell- and chemical-related parameters necessitated a “lumping” approach to assess each category’s contribution to prediction variance. This method may obscure the detailed effects of individual parameters and increase residual contributions to variance; notably, the residual contribution for predicting chemical fractions in cells exceeded 50% (Figure 3). Lastly, the limited number of chemicals (*n* = 15, with poorer coverage of chemical space) examined regarding the impact of considering unbound plasma and media concentrations on in vitro–in vivo concordance resulted from data constraints related to the need for better overlap among chemicals tested in in vitro assays, chemicals for which TK parameters are available, and chemicals for which regulatory in vivo dose–response data are available for determining PODs in the form of BMA BMD_H_. Moreover, this comparison is further limited due to the in vivo data being derived from experimental animal studies, whereas much of the in vitro data were derived from human cells.

Future research directions should focus on addressing these limitations by gathering more experimental data for model performance comparisons, particularly for measured cellular concentrations and comprehensive datasets for assessing the effects of in vitro and in vivo bioavailability adjustments on IVIVE. The review by Proença et al. [13] indicated that many in vitro mass balance models were developed primarily for traditional systems involving monolayers of cells in static media and plastic labware. However, there is an increasing demand for novel in vitro models, such as microphysiological systems (MPSs), which incorporate fluidic microenvironments and multiple cell layers and types to better mimic in vivo conditions, thereby enhancing IVIVE, toxicity assessments, and pharmaceutical development [13,48,49,50].

Consequently, there is a growing need for new in vitro mass balance models tailored for MPSs, particularly given recognized issues related to material absorption in MPSs [51]. Unlike traditional systems, MPSs often utilize various materials such as polydimethylsiloxan, poly (methyl methacrylate), polycarbonate, polystyrene, and polysulfone, sometimes along with coatings that can influence partitioning to labware itself [52,53]. This necessitates the collection of partitioning coefficients for water with other materials, rather than the conventional water–plastic coefficients. Additionally, the complex geometric characteristics of MPSs—including multiple wells and microchannels—result in increased media contact with devices and consequently higher mass transfer rates [54]. Moreover, while chemical distribution to cells in traditional in vitro systems is typically characterized by specific partition coefficients, cellular distributions in MPSs, which often involve multiple cell types and layers, require characterization based on intricate permeability and diffusion dynamics [55]. Instead of using static medium, the integration of perfused fluidic media in MPSs should also be considered in model development [13].

Based on our findings, we suggest several future directions and practical suggestions for application. First, predictive performance for chemical distributions in media is generally superior to that for distributions in cells, indicating that free media concentrations may be the preferable basis for QIVIVE. Using media may also be preferable because the human TK models are generally considered to be more reliable for predicting plasma concentrations than for predicting tissue-level concentrations. Moreover, because prediction of media levels are relatively accurate, experimental measurements, if feasible, may prioritize cellular concentrations.

Additionally, based on our performance evaluation, the Armitage et al. [19] model appears to be a reasonable first choice due to its relatively high predictive accuracy and low bias, consistent performance across a range of chemical properties, broad applicability across neutral and ionizable chemicals, and overall ease of use. Specifically for cellular distribution of neutral chemicals, the Zaldivar-Comenges et al. [23] model has the best performance, but the amount of validation data is quite limited. Future users of these models for predictions in media should prioritize collection of accurate chemical-related parameters, while those targeting cellular concentrations should also target accurate measurements of cell-related parameters.

However, while in vitro and in vivo bioavailability clearly plays an important role in QIVIVE, as demonstrated by the improvement in concordance when using a constant in vitro POD, evidence that it improves in vivo concordance when combined with in vitro bioactivity assays remains limited. Linking in vivo “free” concentrations in plasma to in vitro “free” concentrations does lead to less conservative extrapolated PODs, and thus, it may be reasonable to wait for stronger evidence of improved accuracy in decision contexts emphasizing sensitivity over specificity. On the other hand, multiple factors unrelated to mass balance models (e.g., quantitative relevance of experimental in vivo and/or in vitro data) may contribute to the lack of strong evidence for improving in vitro and in vivo concordance.

## 5. Conclusions

Overall, our study provides an initial comparison of the predictive performance, parameter sensitivity, and impact on in vitro–in vivo concordance of four in vitro mass balance models. Our evaluation suggests that, among the current generation of mass balance models, there is sufficient accuracy and domain of applicability for their routine use in next-generation chemical risk assessment. Nonetheless, while there is a mechanistic rationale to use “free” concentrations as the basis for QIVIVE, evidence that it improves in vitro to in vivo concordance in PODs remains limited.

## Figures and Tables

**Table 1:** Summary of in vitro mass balance models evaluated.

Model Reference	Applicable In Vitro System	Applicable Chemicals	Model Type	Partitioning Included ^a^				Other Factors/ Processes
				Media	Cell	Lab-Ware	Head-Space	
Fischer et al. [22]	Generic	Neutral/ionized; Non-volatile	Equilibrium partitioning model	√	√ (protein and lipid)			
Armitage et al. [19]	Generic; culture media with serum and monolayer cell	Neutral/ionized; Non-volatile/volatile	Equilibrium partitioning model	√	√	√	√	Solubility
Fisher et al. [16]	Generic	Neutral/ionized; Non-volatile/volatile	Time-dependent model	√	√ (protein, lipid, lysosome and mitochondria)	√	√	Metabolism
Zaldivar-Comenges et al. [23]	Generic	Neutral; Non-volatile/volatile	Time-dependent model	√	√ (protein, lipid and mitochondria)	√	√	Evaporation; abiotic degradation; cell growth

^a^ √ = yes.

**Table 2:** Chemical-related parameters used in four in vitro mass balance models.

Parameters (Abbreviation) [Unit]	Model			
	Fischer et al. [22]	Armitage et al. [19]	Fisher et al. [16]	Zaldivar-Comenges et al. [23]
Molecular weight (MW)		√	√	√
Melting point (MP) [°C]		√	√	
Octanol–water partition coefficient (log K_OW_)	√ ^a^	√	√	√
Air–water partition coefficient (log K_AW_)		√	√	
Solubility (C_SAT,W_) [mg/L]		√		
Salting-out constant (K_salt_)		√ ^b^		
pKa	√ ^a^	√	√	
IOC type		√	√	
Henry’s constant at 37 °C (H37) [Pa × m^3^/mol]				√
Molecular volume (Vb)				√
Distribution ratio at pH 7.4 between bovine serum albumin (BSA) and water (log D_BSA/w_)	√ ^a^			
Distribution ratio at pH 7.4 between phospho-lipid liposomes (lip) and water (log D_lip/w_)	√ ^a^			

^a^ If experimental values for log D_BSA/w_ and log D_lip/w_ are unavailable, predictions based on chemical descriptors from the UFZ-LSER database (http://www.ufz.de/lserd) (accessed on 24 October 2024) are used for neutral chemicals. If the database is also unavailable, log K_OW_ values are utilized to predict distribution ratios. For the IOC, the pKa value is used to correct the log K_OW_, which is then applied for predicting distribution ratios. Further details are provided in the main text. ^b^ Salting-out constant (K_salt_) is predicted by log K_OW_: K_salt_ = 0.04 × log K_OW_ + 0.114 (sourced from EAS-E Suite platform). Note: √ = parameter included.

**Table 3:** Data used for model performance comparisons.

Dataset	Chemical (s) ^a^	Cell Type (s) ^b^	Endpoint Measurement
Ratio of free to nominal concentration			
Huchthausen et al. [24]	Neutral and IOC (*n* = 12)	MCF7, HEK293H	Measured freely dissolved IC10 conc., then obtain ratio
Tanneberger et al. [25]	Neutral and IOC (*n* = 27)	Fish RTgill-W1	Ratio of conc. in medium at the end and beginning of experiments (C_24h_/C_0h_)
Schug et al. [26]	Neutral organics (*n* = 9)	Fish RTgutGC	Ratio of conc. in medium at the end and beginning of experiments (C_24h_/C_0h_)
Nicol et al. [27]	Neutral and IOC (*n* = 30)	No cell	Ratio of conc. in buffer and in total matrix. These measurements were performed using a Rapid Equilibrium Dialysis (RED) plate
Valdiviezo et al. [28]	Pesticides (*n* = 20)	No cell	Ratio of response ratios measured from free media and initial exposure media
Blanchette et al. [29]	Neutral and IOC (*n* = 30)	No cell	Ratio of response ratios measured from free media and initial exposure media
This study	PFAS (*n* = 14)	No cell	Ratio of response ratios measured from free media and initial exposure media
Amount of chemicals in media and/or cells			
Bellwon et al. [30]	Cyclosporine A (*n* = 1)	PRH, PHH, HepaRG	Measured chemical amount in cell lysate and media at multiple timepoint (≤24 h)
Broeders et al. [31]	Chlorpromazine (*n* = 1)	Balb/c 3T3, Caco-2, HepaRG	Measured chemical distribution % in cells and medium at 48 h (Balb/c 3T3 and Caco-2 cells) or 72 h (HepaRG cells)
Broeders et al. [32]	Chlorpromazine (*n* = 1)	PRH, PHH, HepaRG	Measured chemical amount in cell lysate and media at multiple timepoint (≤24 h)
Kodavanti et al. [33]	PBDEs (*n* = 3)	Rat cerebellar granule cell	Measured chemical amount in cell lysate at multiple timepoint (≤24 h)
Pomponio et al. [34]	Amiodarone (*n* = 1)	PHH, HepaRG	Measured chemical amount in cell lysate at multiple timepoint (≤24 h)
Truisi et al. [35]	Ibuprofen (*n* = 1)	PRH, PHH, HepaRG	Measured chemical amount in cell lysate and media at multiple timepoint (≤24 h)

^a^ Chemical-related parameters were summarized in Supplemental Table S2. ^b^ Cell-related parameters were summarized in Supplemental Table S3. Abbreviations: IOC: Ionizable organic chemical; PFAS: Per- and polyfluoroalkyl substances; PBDEs: Polybrominated Diphenyl Ethers; PRH: Primary rat hepatocyte; PHH: Primary human hepatocyte.

## Data Availability

The data and model executing codes presented in the study are openly available in GitHub repository at https://github.com/hsingchiehlin/Invitro_mass_balance_models.

## Supplementary Materials

The following supporting information can be downloaded at: https://www.mdpi.com/article/10.3390/toxics13060439/s1, Figure S1: Satter plots of relationships among log KOW, solubility and vaper pressure for unique 116 chemicals used to model performance comparison, 15 chemicals used to model application, and the chemicals in the Collaborative Estrogen Receptor Activity Prediction Project3 (CERAPP), as well as relationships among top three principal component after conducting PCA analysis; Figure S2: Histograms of errors of comparing observed (A) ratios of free to nominal concentration, (B) amounts of chemicals in media, and (C) in cells and their corresponding predictions by using four in vitro mass balance models; Figure S3: Scatter plots comparing observed free fraction in media, amounts of chemicals in media, or cells and their corresponding predictions by using four in vitro mass balance models for only neutral chemicals and only non-neutral chemicals; Figure S4. Scatter plots comparing observed free fraction in media and their corresponding predictions by using four in vitro mass balance models for all the datapoints in Figure 3 (top row) and the comparison with removing the datapoints with mass balance issue; Figure S5: Relationships of chemicals properties, including nominal concentration (Conc), Henry’s constant (H37), solubility, KAW, KOW, melting point (MP), molecule weight (MW), and pKa and errors of observed ratios of free to nominal concentration and their predictions; Figure S6: Relationships of chemicals properties, including nominal concentration (Conc), Henry’s constant (H37), solubility, KAW, KOW, melting point (MP), molecule weight (MW), and pKa and errors of observed fraction of chemical amount in cell and their predictions; Figure S7: Simulated ratios of free to nominal concentration by 4 mass balance models with 13 cell types, 2 types of labware (96- and 384-well), and 2 percentages of FBS (2% and 20%) for 116 chemicals; Figure S8: Simulated fraction of chemicals in cells by 4 mass balance models with 13 cell types, 2 types of labware (96- and 384-well), and 2 percentages of FBS (2% and 20%) for 116 chemicals; Figure S9: Differences of ratios of free to nominal concentration by using Armitage et al. (2021) [19] models with default cell and by four mass balance models with other 12 cell types for 116 chemicals; Figure S10: Differences of fraction of chemicals in cells by using Armitage et al. (2021) [19] models with default cell and by four mass balance models with other 12 cell types for 116 chemicals; Figure S11: Bar chart summarizing the Spearman ρ, Pearson r, mean absolute error (MAE), and mean error (ME) values for comparing oral equivalent dose from in vivo and in vitro POD under both the “without fraction unbound adjustment” and “with fraction unbound adjustment” conditions; Figure S12. Histogram of ratios of predicted fub, media by four in vitro mass balance model to fub, plasma for 15 chemicals used for model application and 68 chemicals from sensitivity analysis; Table S1: Datasets used for model performance comparisons; Table S2: Chemical-related parameters applied to the four in vitro mass balance models; Table S3: Cell-related parameters used for model comparison and sensitivity analysis; Table S4: Observed and predicted value used for model performance comparisons; Table S5: Dataset used to conduct PCA analysis; Table S6: Details of pKa prediction for 116 chemicals used for model performance and 15 chemicals for model application; Table S7: Detailed information and predictions of parameters used for Fischer et al. (2017) [22] model; Table S8: Dimension of labwares used in this study; Table S9: List of the in vivo, in vitro PODs from Lu et al. (2024) [41], C_SS_, *f*_ub,plasma_ from *httk* database, and *f*_ub,media_ predicted by four mass balance models; Table S10: Summary of the slopes and determination coefficients for Figures S5 and S6; Table S11: Details of Spearman *ρ*, Pearson *r*, mean absolute error (MAE), and mean error (ME) value for comparing oral equivalent dose from in vivo and in vitro POD with/without adjustment by using fraction unbound in plasma and in media predicted by four mass balance models [28,36,56,57,58,59,60,61,62,63,64,65,66,67,68,69,70,71,72].

## Abbreviations

The following abbreviations are used in this manuscript:

QIVIVE	Quantitative In Vitro to In Vivo Extrapolation
Tox21	Toxicity Testing in the 21st Century
HTS	High-Throughput Screening
BED	Biologically Effective Dose
PBK	Physiologically Based Kinetics
iPSCs	Induced Pluripotent Stem Cells
POD	Point of Departure
IOC	Ionizable Organic Chemical
PFAS	Per- and Polyfluoroalkyl Substances
PBDEs	Polybrominated Diphenyl Ethers
PRH	Primary Rat Hepatocyte
PHH	Primary Human Hepatocyte
RED	Rapid Equilibrium Dialysis
FBS	Fetal Bovine Serum
CERAPP	Collaborative Estrogen Receptor Activity Prediction Project
PCA	Principal Component Analysis
MAE	Mean Absolute Error
ME	Mean Error
MW	Molecular Weight
MP	Melting Point
OED	Oral Equivalent Dose
iTTC	Internal Threshold of Toxicological Concern
