# Supplementary material for: Comparative Analysis of Chemical Distribution Models for Quantitative In Vitro to In Vivo Extrapolation
Source: Toxics. 2025 May 26;13(6):439. doi: 10.3390/toxics13060439 (PMC12196727; doi:10.3390/toxics13060439)
Supplement: Supplementary file 1 [file toxics-13-00439-s001.zip › toxics-3597042-supplementary.pdf]

toxics

MDPI

Supplementary Materials: Comparative Analysis of Chemical Distribution Models for Quantitative *In Vitro* to *In Vivo* Extrapolation

Hsing-Chieh Lin, Lucie C. Ford, Ivan Rusyn, Weihsueh A. Chiu

Media Protein Binding Assay and Analysis for PFAS

The media used for the protein binding analysis, consisted of iCell Cardiomyocytes maintenance medium (Cat # M1003, FujiFilm CDI, Madison, Wisconsin), which contains 10% FBS and supplemented with 1:500 (v/v) penicillin-streptomycin (Cat# 15140-122 100ML, Gibco, Waltham, MA). Two sets of samples were prepared to determine the unbound fraction of the PFAS. Firstly, for the middle layer media analysis, 2 mL of media was aliquoted for each concentration (1 and 10 μM) and chemical of interest, the chemical stocks (prepared in 100% DMSO) were then spiked into the media.[28] The spiked media aliquots were mixed at 550 RPM for 1 hour at 37°C. After the incubation period, 300 μL of the spiked media samples was transferred to an ultracentrifuge tube, transferring 3 aliquots per sample, the tubes were weighed and adjusted to be within a difference of 0.0010g. The ultracentrifuge tubes were placed on the Optima MAX-XP Ultracentrifuge (Cat#: 393315; Beckman Coulter, Brea, CA) according to their weight balance. The samples were then spun at 90,000 RPM for 4.5 hours at 4°C. The tubes were then collected and 100 μL of the middle layer was transferred to a 1.5 mL microcentrifuge tube containing 200 μL of chilled acetonitrile with the internal standard and vortexed. The samples were then spun down at 12,000 RCF for 10 minutes, the supernatant was then transferred to a new 1.5 mL microcentrifuge tube and placed in the Savant SpeedVac (Cat# SPD1030-230, Thermo Scientific, Waltham, MA) until dry. Once dry, the samples were reconstituted in 100 μL of aqueous mobile phase (water containing 5mM ammonium acetate), vortexed and transferred to LC-MS/MS vials. Similarly, for the initial condition analysis each chemical and concentration was spiked into 2 mL of media, the samples were then vortexed, and 100 μL was transferred into 3 individual 1.5 mL microcentrifuge tubes containing chilled acetonitrile with the internal standard. The 3 replicates for each condition were then vortexed and centrifuged at 12,000 RCF for 10 minutes. The supernatant was collected and transferred to a new 1.5 mL microcentrifuge tube and placed in the Savant SpeedVac (Thermo Scientific). Once dry, the samples were reconstituted with 100 μL of the aqueous mobile phase (water containing 5mM of Ammonium Acetate), they were vortexed and transferred to a LC-MS/MS vial. The samples were then run on the LC-MS/MS for data acquisition and quantification using an Agilent (Agilent, Santa Clara, CA) 1260 Infinity II High Speed Pump coupled with a 6470 triple quadrupole MS with negative mode electron spray ionization. The analytical details for the PFAS are detailed in [56]. After the data was collected the % Free was determined by using the following ratio:

$$F_{ub}(\%free) = \frac{ISTD\ resp.ratio\ (middle\ layer)}{ISTD\ resp.ratio\ (Initial)}$$

Supplementary figures

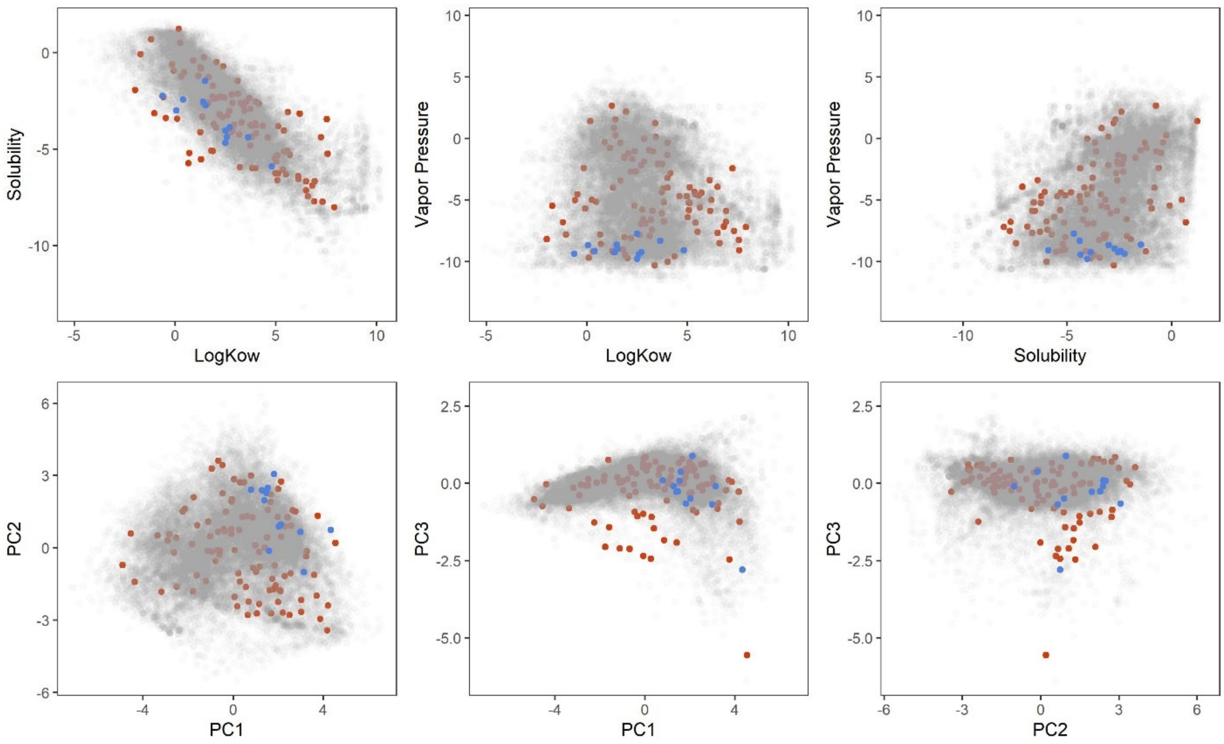

Figure S1. Satter plots of (upper 3 panels) relationships among log KOW, solubility and vaper pressure for unique 116 chemicals used to model performance comparison (red points), 15 chemicals used to model application (blue points), and the chemicals in the Collaborative Estrogen Receptor Activity Prediction Project [36] (CERAPP; gray points; n = 24,955), as well as (lower 3 panels) relationships among top three principal component after conducting PCA analysis.

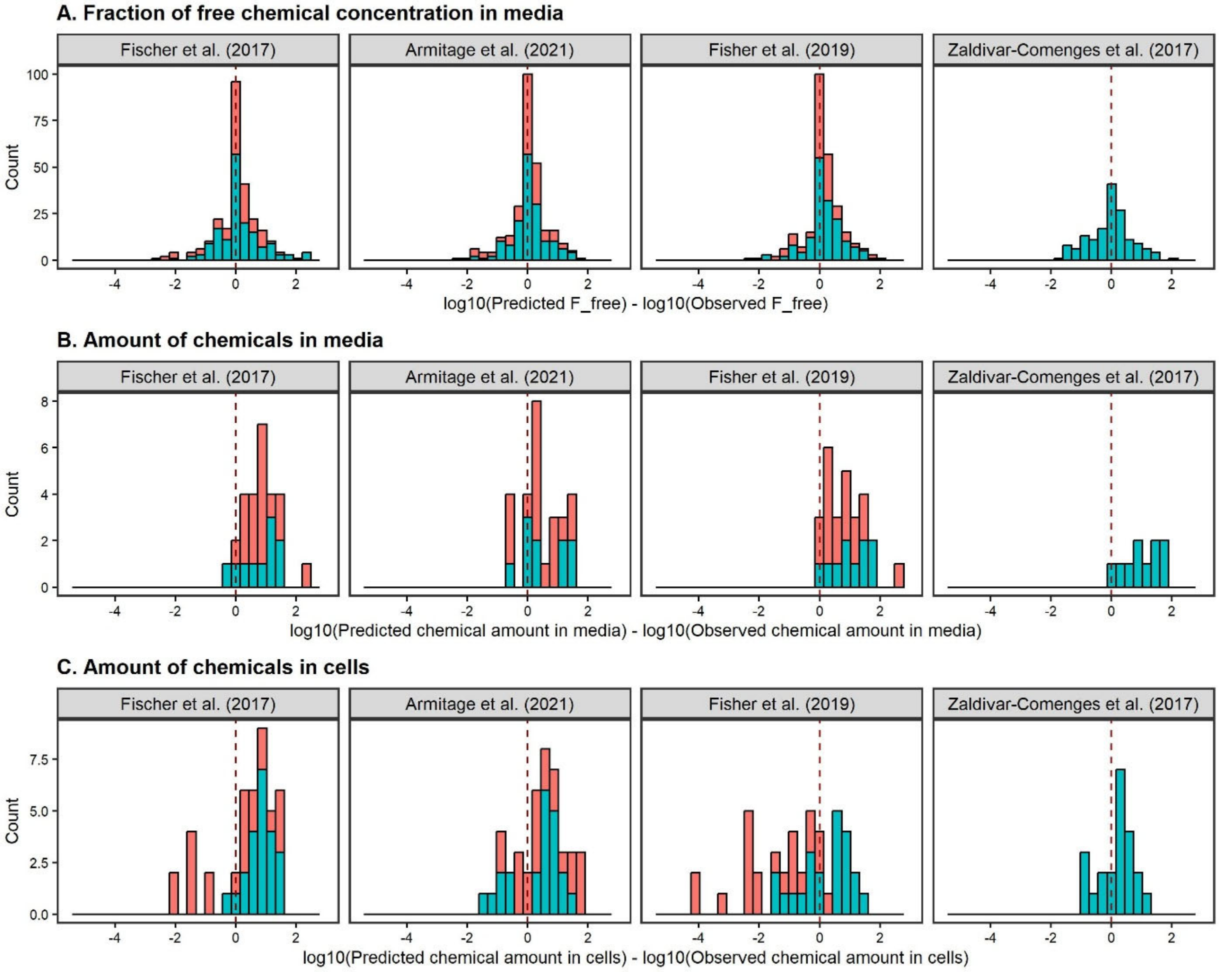

Figure S2. Histograms of errors of comparing observed (A) ratios of free to nominal concentration, (B) amounts of chemicals in media and (C) in cells and their corresponding predictions by using four in vitro mass balance models. Blue bars represent neutral chemicals, while red bars represent non-neutral chemicals.

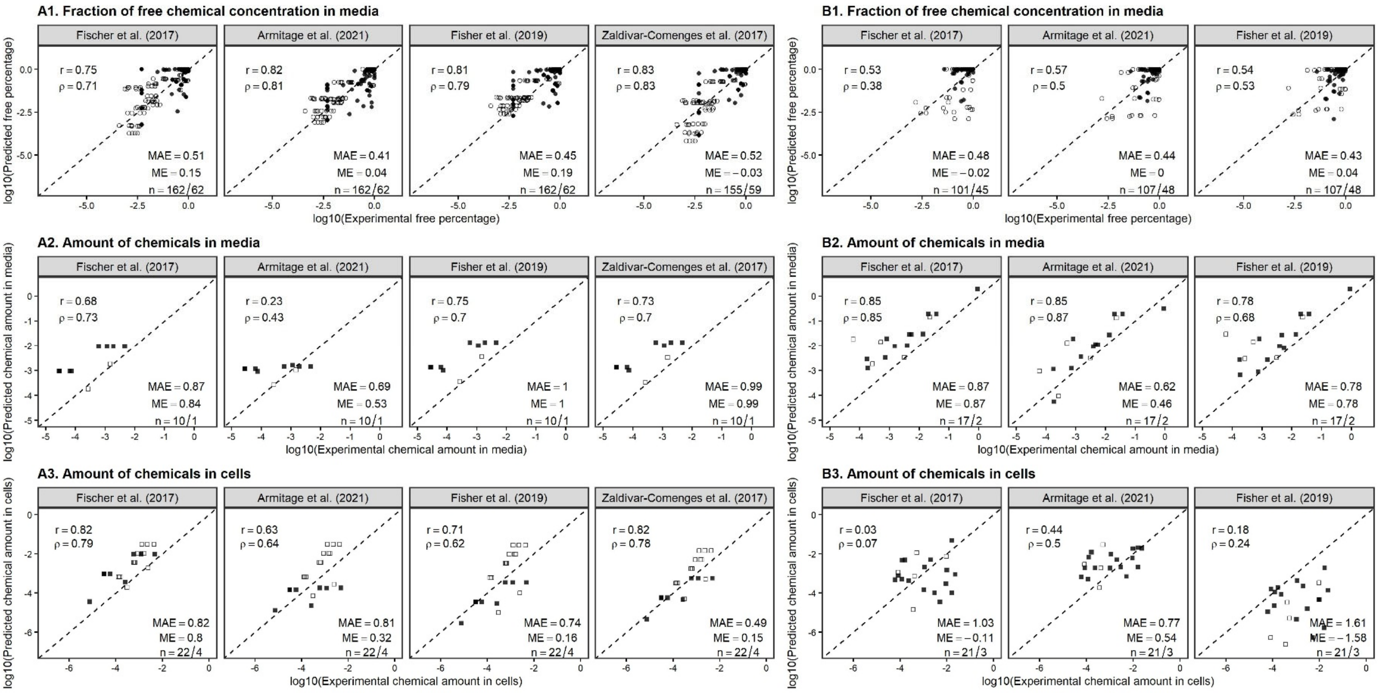

Figure S3. Scatter plots comparing observed free fraction in media (ratios of free to nominal concentration) (top row), amounts of chemicals in media (middle row), or cells (bottom row) and their corresponding predictions by using four in vitro mass balance models for (A) only neutral chemicals and (B) only non-neutral chemicals. For free fraction (top row), the filled points represent the data sourced from the literature, the open points represent experimental data from our previous publications. For amounts in media (middle row) or cells (bottom row), the filled points represent the experimental data tested using human cells, and open points represent experimental data tested using non-human cells. Each graph shows correlation values and prediction errors, as well as the number of chemicals and data points included in each scatter plot (n = number of datapoints/number of chemical).

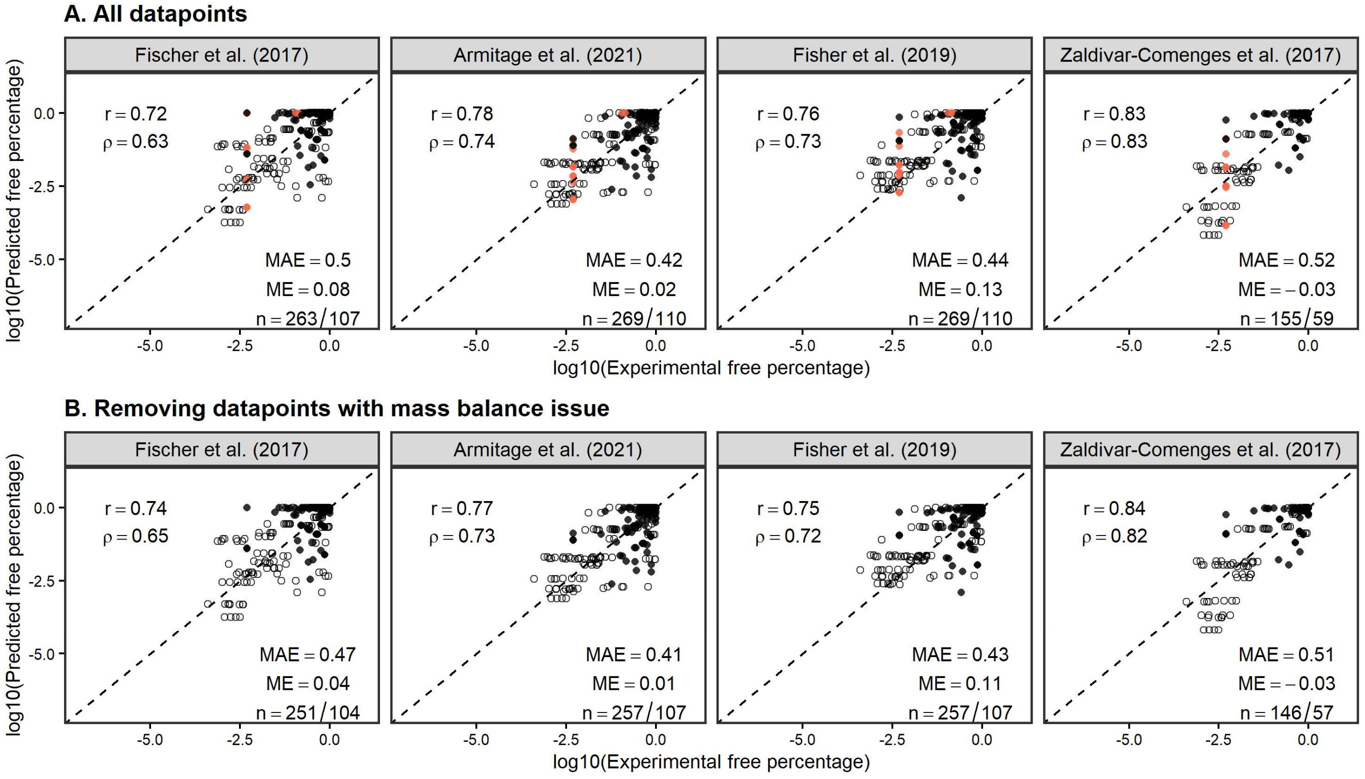

Figure S4. Scatter plots comparing observed free fraction in media (ratios of free to nominal concentration) and their corresponding predictions by using four in vitro mass balance models for (A) all the datapoints in Figure 3 (top row) and (B) removing the datapoints with mass balance issue. In (A), the red points represent the data with mass balance issue. Each graph shows correlation values and prediction errors, as well as the number of chemicals and data points included in each scatter plot (n = number of datapoints/number of chemical).

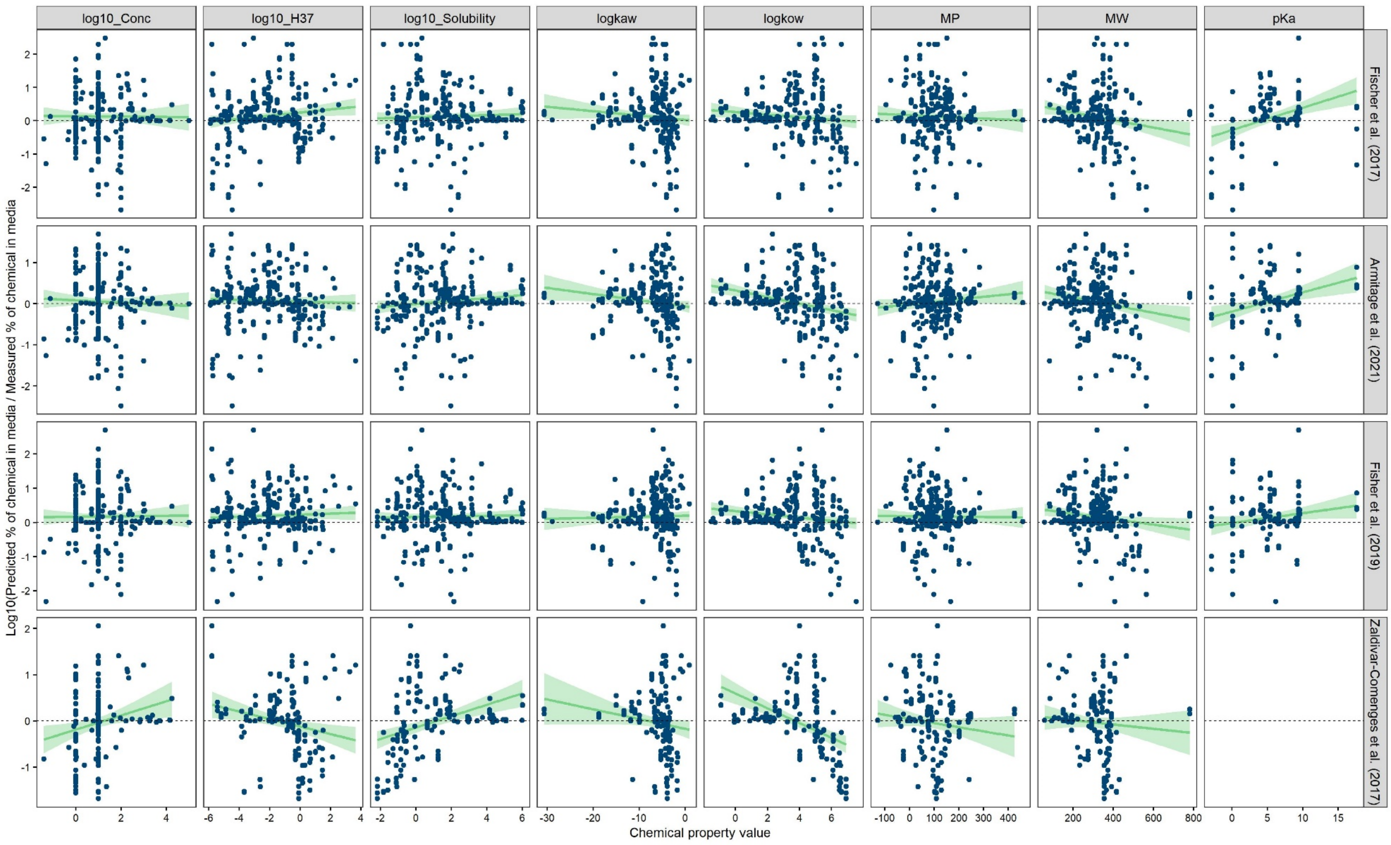

Figure S5. Relationships of chemicals properties, including nominal concentration (Conc), Henry's constant (H37), solubility, KAW, KOW, melting point (MP), molecule weight (MW), and pKa and errors of observed ratios of free to nominal concentration and their predictions.

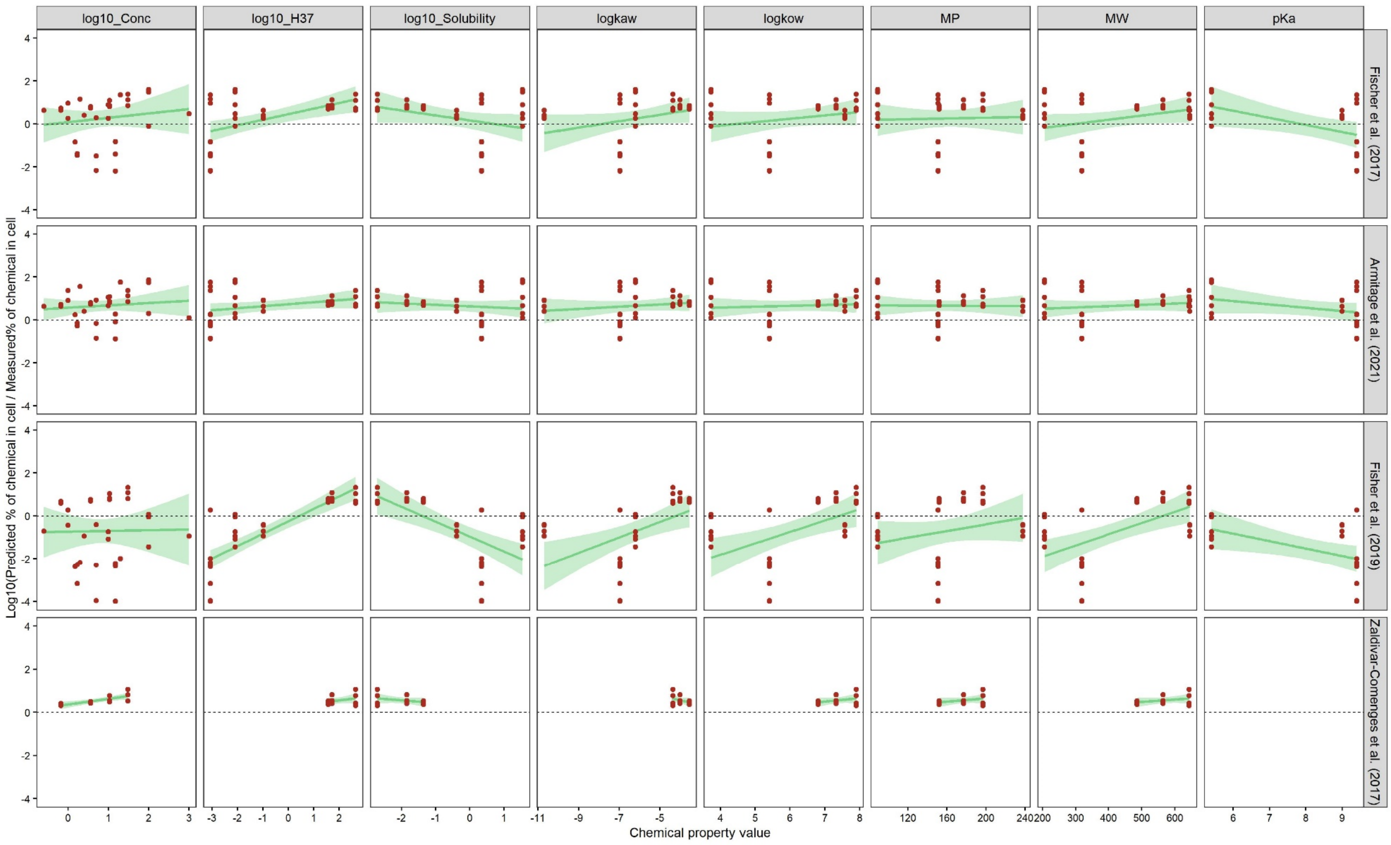

Figure S6. Relationships of chemicals properties, including nominal concentration (Conc), Henry's constant (H37), solubility, KAW, KOW, melting point (MP), molecule weight (MW) , and pKa and errors of observed fraction of chemical amount in cell and their predictions.

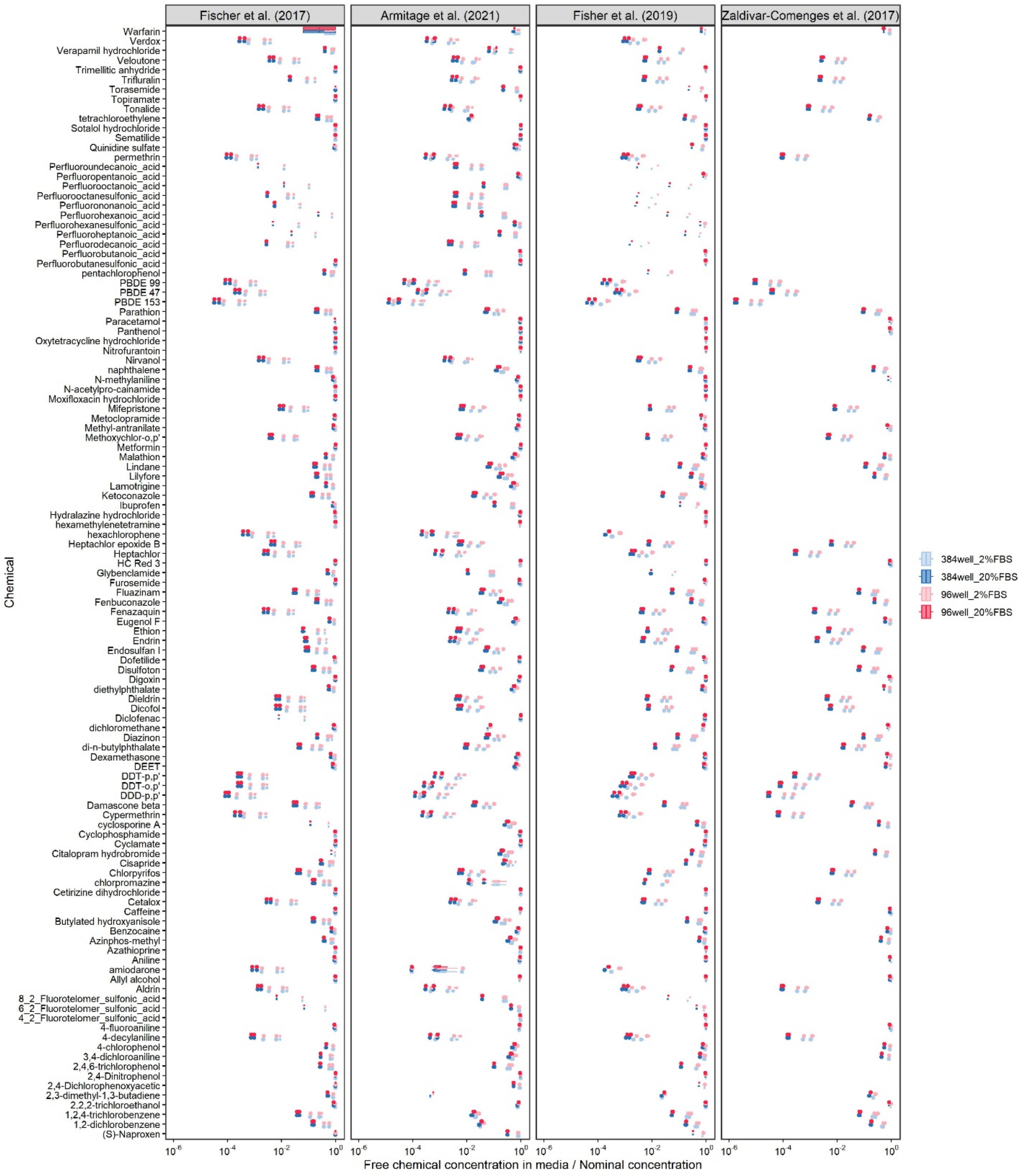

Figure S7. Simulated ratios of free to nominal concentration by 4 mass balance models with 13 cell types, 2 types of labware (96- and 384-well), and 2 percentages of FBS (2% and 20%) for 116 chemicals. Each box-plot represents variation across 13 cell types.

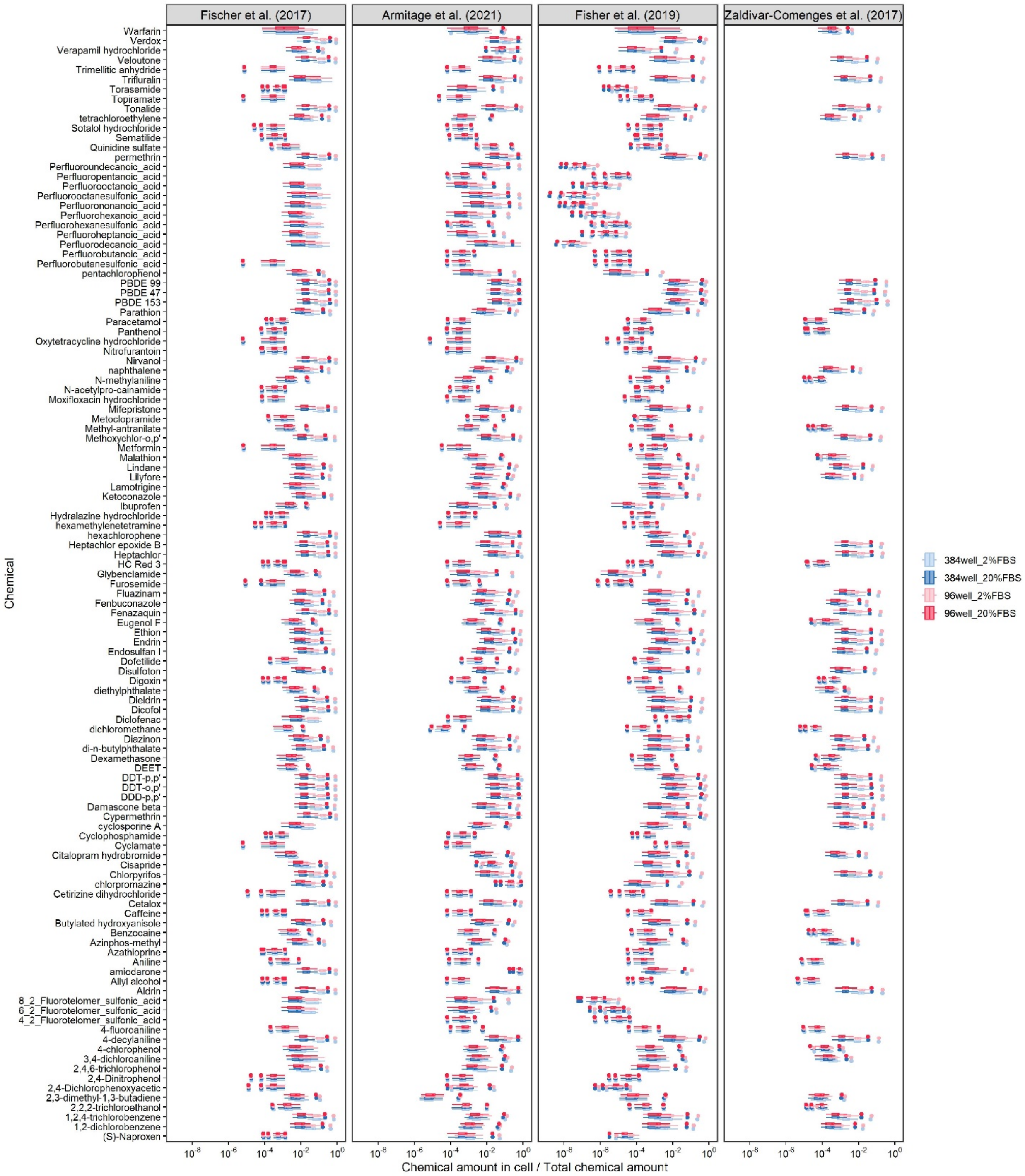

Figure S8. Simulated fraction of chemicals in cells by 4 mass balance models with 13 cell types, 2 types of labware (96- and 384-well), and 2 percentages of FBS (2% and 20%) for 116 chemicals. Each box-plot represents variation across 13 cell types.

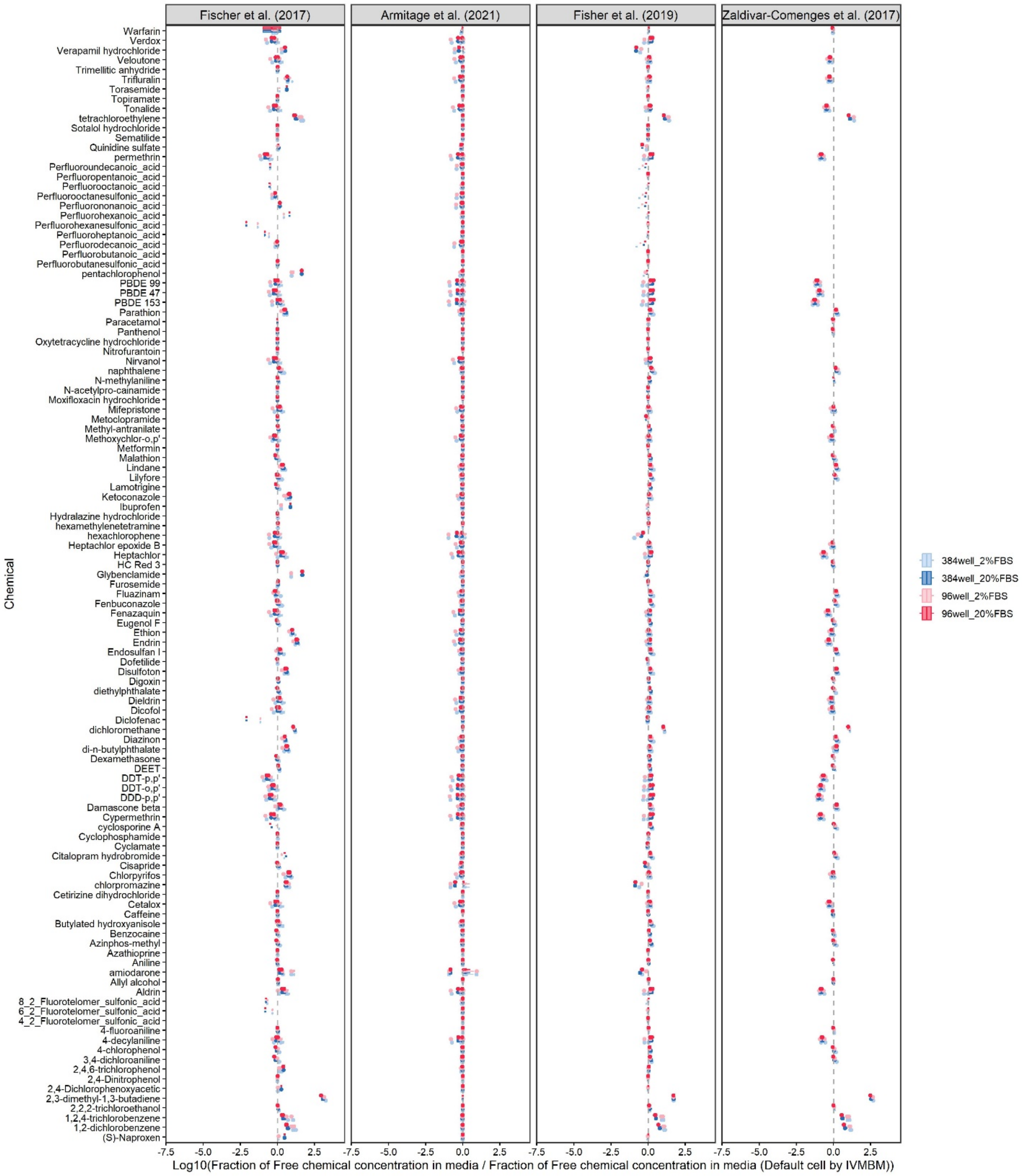

Figure S9. Differences of ratios of free to nominal concentration by using Armitage et al. (2021) models with default cell and by four mass balance models with other 12 cell types for 116 chemicals. Each box-plot represents variation across 12 cell types.

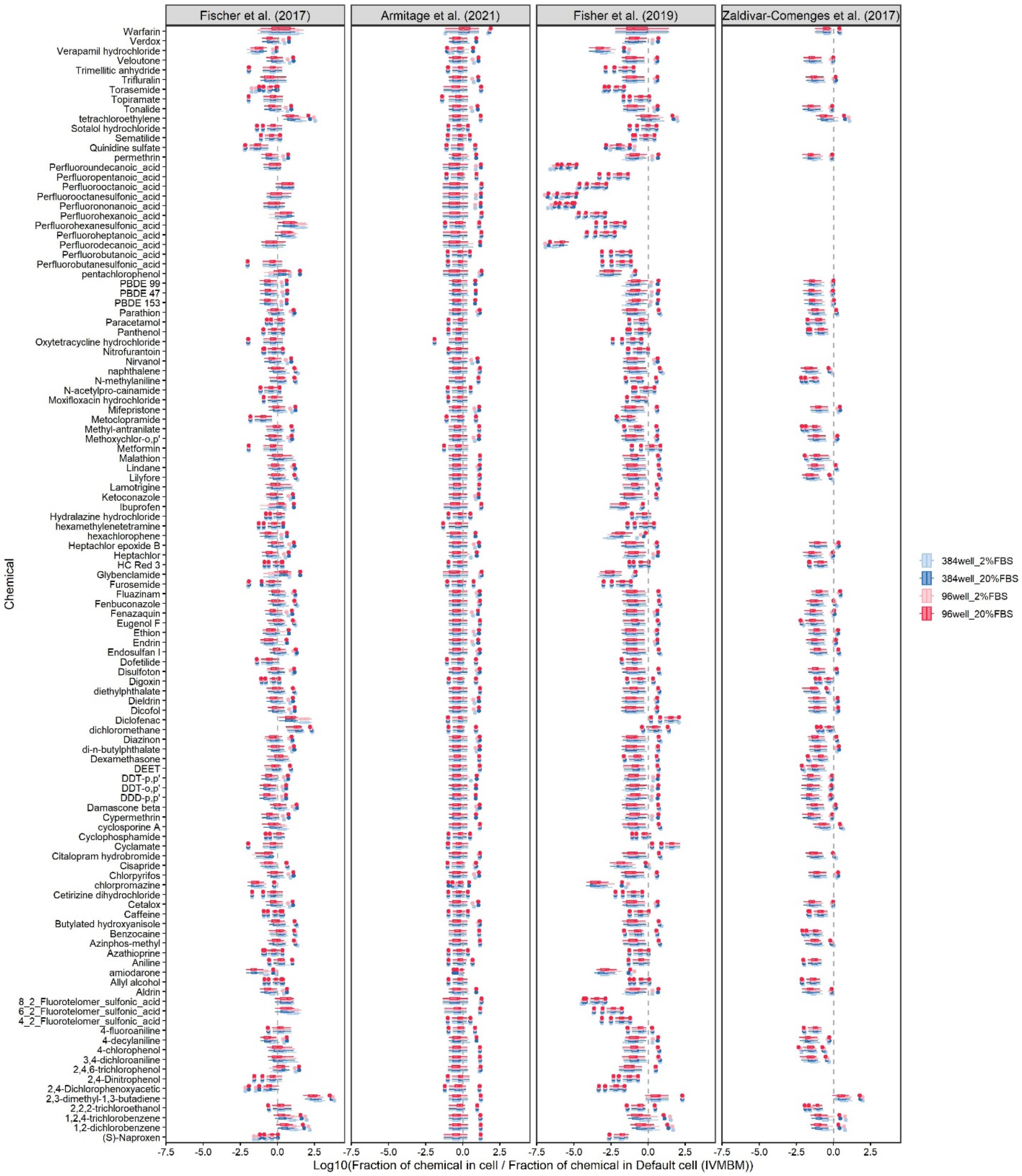

Figure S10. Differences of fraction of chemicals in cells by using Armitage et al. (2021) models with default cell and by four mass balance models with other 12 cell types for 116 chemicals. Each box-plot represents variation across 12 cell types.

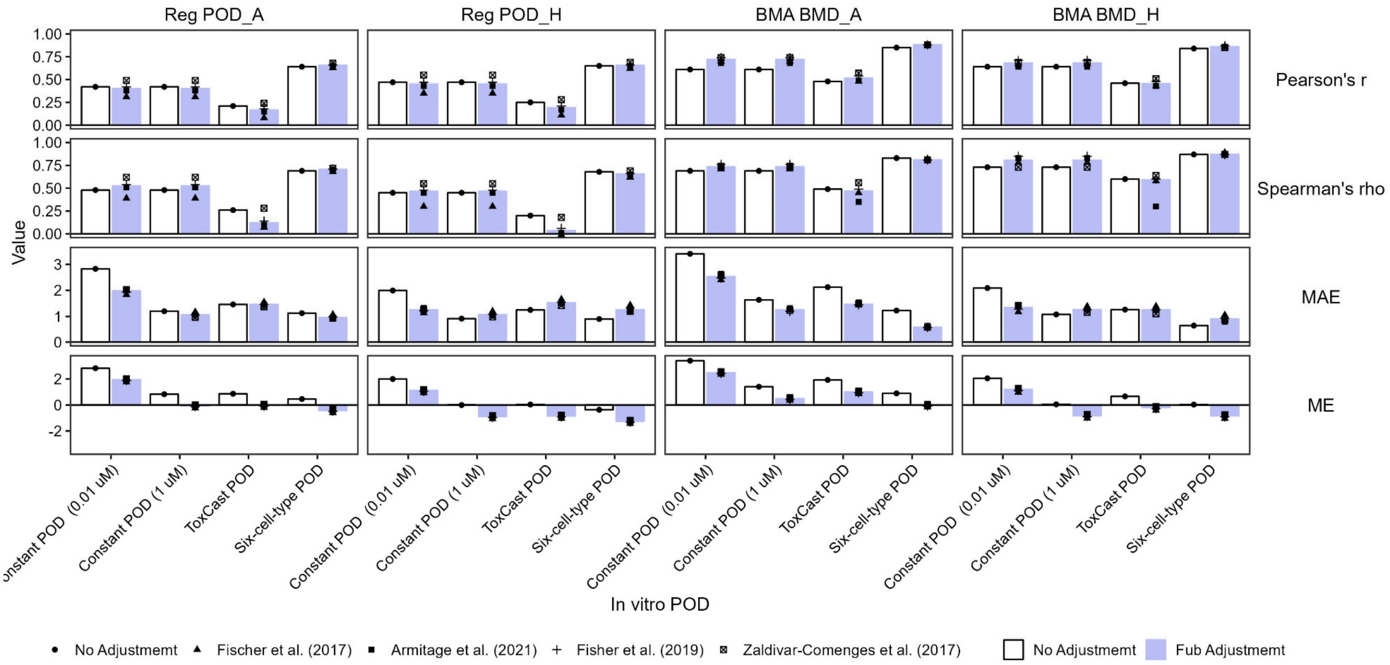

Figure S11. Bar chart summarizing the Spearman ϱ, Pearson r, mean absolute error (MAE), and mean error (ME) values for comparing oral equivalent dose from in vivo and in vitro POD under both the "without fraction unbound adjustment" and "with fraction unbound adjustment" conditions. The points represent the values for each metric (Spearman ϱ, Pearson r, MAE, and ME) calculated using four different in vitro mass balance models, as well as the fraction unbound in plasma from the httk database. The blue bars represent the mean values across the four models for the fraction unbound adjustment. Details of the in vivo and in vitro POD are provided in Tables S9.

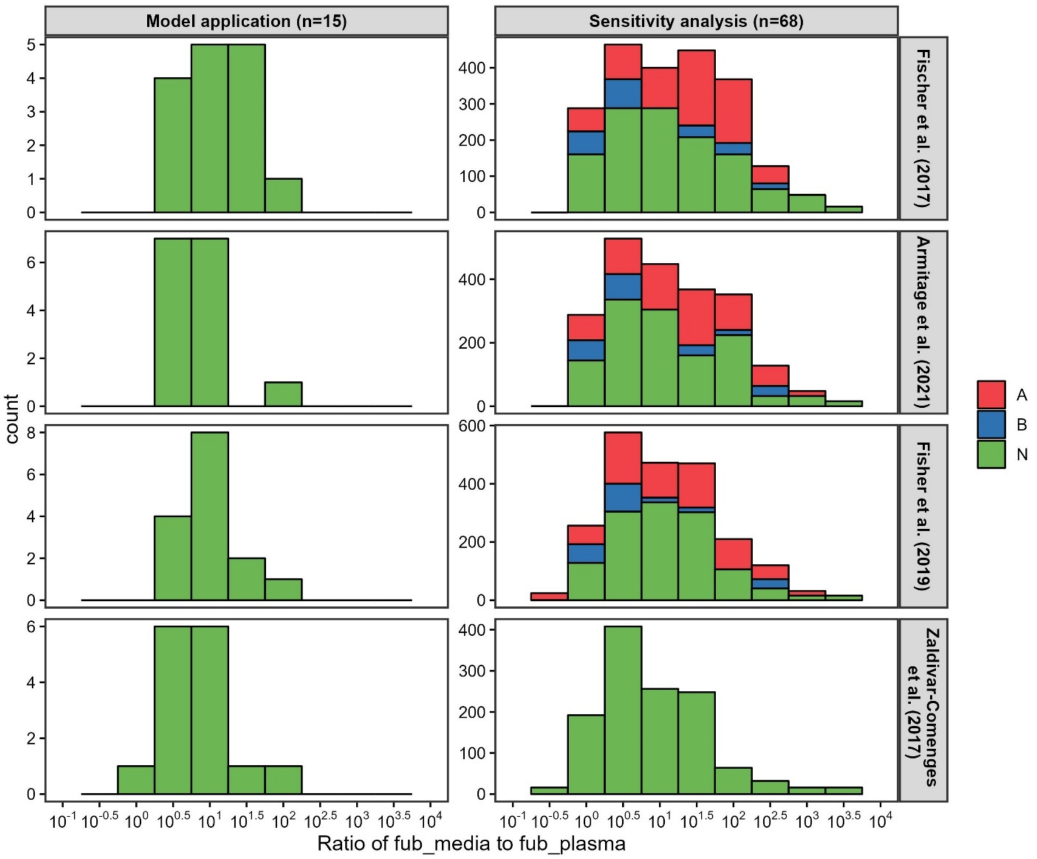

Figure S12. Histogram of ratios of predicted fub, media by four in vitro mass balance model to fub, plasma for 15 chemicals used for model application and 68 chemicals from sensitivity analysis.
